# Impact of Porosity and Stiffness of 3D Printed Polycaprolactone Scaffolds on Osteogenic Differentiation of Human Mesenchymal Stromal Cells and Activation of Dendritic Cells

**DOI:** 10.1021/acsbiomaterials.4c01108

**Published:** 2024-11-01

**Authors:** Mehmet Serhat Aydin, Nora Marek, Theo Luciani, Samih Mohamed-Ahmed, Bodil Lund, Cecilie Gjerde, Kamal Mustafa, Salwa Suliman, Ahmad Rashad

**Affiliations:** † Center of Translational Oral Research (TOR), Department of Clinical Dentistry, University of Bergen, Bergen 5009, Norway; ‡ Department of Dental Medicine, Karolinska Institute, Stockholm 17177, Sweden; § Medical Unit of Plastic Surgery and Oral and Maxillofacial Surgery, Karolinska University Hospital, Stockholm 17177, Sweden; ∥ Bioengineering Graduate Program, Aerospace and Mechanical Engineering, University of Notre Dame, Notre Dame, Indiana 46556, United States

**Keywords:** Salt leaching, Nonsolvent induced phase separation, Microporosity, Degradation, Bone tissue engineering, Immune-mediated bone regeneration

## Abstract

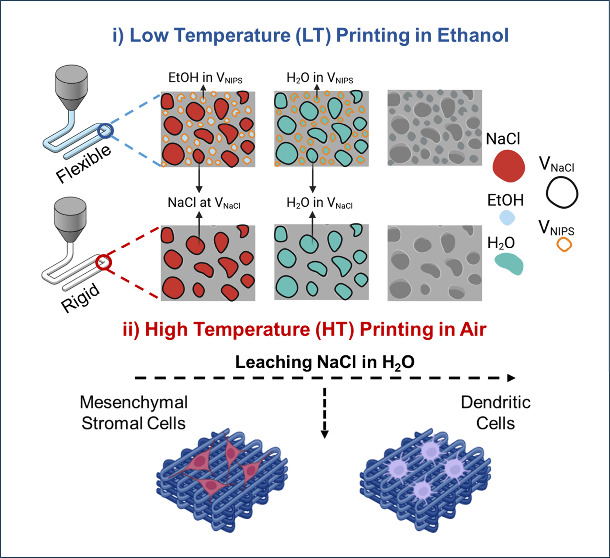

Despite the potential of extrusion-based printing of thermoplastic polymers in bone tissue engineering, the inherent nonporous stiff nature of the printed filaments may elicit immune responses that influence bone regeneration. In this study, bone scaffolds made of polycaprolactone (PCL) filaments with different internal microporosity and stiffness was 3D-printed. It was achieved by combining three fabrication techniques, salt leaching and 3D printing at either low or high temperatures (LT/HT) with or without nonsolvent induced phase separation (NIPS). Printing PCL at HT resulted in stiff scaffolds (modulus of elasticity (E): 403 ± 19 MPa and strain: 6.6 ± 0.1%), while NIPS-based printing at LT produced less stiff and highly flexible scaffolds (E: 53 ± 10 MPa and strain: 435 ± 105%). Moreover, the introduction of porosity by salt leaching in the printed filaments significantly changed the mechanical properties and degradation rate of the scaffolds. Furthermore, this study aimed to show how these variations influence proliferation and osteogenic differentiation of human bone marrow-derived mesenchymal stromal cells (hBMSC) and the maturation and activation of human monocyte-derived dendritic cells (Mo-DC). The cytocompatibility of the printed scaffolds was confirmed by live–dead imaging, metabolic activity measurement, and the continuous proliferation of hBMSC over 14 days. While all scaffolds facilitated the expression of osteogenic markers (RUNX2 and Collagen I) from hBMSC as detected through immunofluorescence staining, the variation in porosity and stiffness notably influenced the early and late mineralization. Furthermore, the flexible LT scaffolds, with porosity induced by NIPS and salt leaching, stimulated Mo-DC to adopt a pro-inflammatory phenotype marked by a significant increase in the expression of IL1B and TNF genes, alongside decreased expression of anti-inflammatory markers, IL10 and TGF1B. Altogether, the results of the current study demonstrate the importance of tailoring porosity and stiffness of PCL scaffolds to direct their biological performance toward a more immune-mediated bone healing process.

## Introduction

In bone tissue engineering, porous scaffolds are designed to instruct not only the osteogenic differentiation of mesenchymal stromal cells but also immune cells, thus initiating an immune-mediated regeneration.^1−3^ For the same material chemistry, scaffold porosity, pore size, pore geometry, and pore interconnectivity play a significant role that profoundly dictate the mechanical and biological functionalities of scaffolds.^4^ Although, there is a wide range of optimal porosity reported for *in vitro* bone tissue engineering, there is growing evidence that a pore size larger than 300 μm is needed for *in vivo* vascularization and bone formation.^5−8^ Interconnected large pores (macroporosity) allow for oxygen diffusion, nutrition exchange, waste removal, cell migration, and tissue ingrowth through the scaffold, thus supporting angiogenesis and direct osteogenesis.^9^ The presence of smaller pores (microporosity) enhances the specific surface area and provides more sites for protein adsorption, which facilitates stromal cell attachment and osteogenic differentiation.^9^ Moreover, scaffold porosity was reported to have profound influence on the phenotype and function of immune cells such as macrophages and dendritic cells (DC).^2,10^ For example, maturation of DC was reported to increase with decreasing pore size when cells were cultivated on hydrophilic poly(2-Hydroxyethyl methacrylate) (pHEMA) and hydrophobic polydimethylsiloxane (PDMS) scaffolds with 20, 40, 90 μm pore sizes.^10^

In scaffold-driven bone regeneration, the porosity influences not only the scaffold’s biological properties but also its mechanical performance and degradation profile. Increased porosity within a scaffold can lead to reduced stiffness compared to a denser counterpart.^11^ Depending on the chemical structure of polymeric scaffolds, the degradation rate can be increased or decreased as a function of porosity.^12^ As a result, this affects the overall biological properties of the scaffold. Soft polymeric scaffolds with 60% porosity were reported to facilitate the proliferation of mesenchymal stromal cells (MSC), whereas stiff scaffolds (with 20% porosity) favored osteogenic differentiation. With regards to immune cells, macrophages were more driven by the high stiffness of these scaffolds to polarize toward the pro-inflammatory M1 phenotype.^3^ It was suggested that stiffness regulates the polarization of macrophages *in vitro* and *in vivo* via the NF-κB signaling pathway.^13,14^ In terms of dendritic cells (DC), when cultured on PDMS with high stiffness, they exhibited increased proliferation, activation, and cytokine production compared to those cultured at physiological resting stiffness. This effect was suggested to be mediated through the pathway of Hippo-signaling.^10^

Polycaprolactone (PCL) is one of the most common FDA approved thermoplastic polymers used for scaffold fabrication in bone tissue engineering.^15,16^ There are several techniques available to incorporate porosity into thermoplastic scaffolds, such as solvent casting and particulate leaching (salt leaching), gas foaming, phase separation, freeze-drying, and electrospinning.^17^ However, these conventional methods often lack precise control over scaffold porosity.^18^ In contrast, 3D extrusion-based printing offers a highly automated fabrication process that can produce patient-specific scaffolds with controlled large-scale porosity.^18^ It has been reported that the extrusion-based 3D printing of PCL at high temperatures (around 100 °C) can produce scaffolds with controlled porosity and customized shapes.^19^ However, a drawback of this approach is the inherent nonporous solid and rigid nature of the printed filaments. It is still a challenge to produce 3D-printed PCL filaments with multiscale porosity and tailored mechanical properties that can guide not only human bone marrow-derived MSC (hBMSC) but also modulate immune cells such as DC.

To overcome this limitation, we, in the current study, introduced a new approach for the fabrication of PCL scaffolds based on a combination of extrusion-based 3D printing, salt leaching, and nonsolvent induced phase separation (NIPS) techniques. This approach facilitated the production of highly porous and highly flexible PCL scaffolds printed at a low temperature (30 °C). To produce porous but comparatively stiffer scaffolds, salt leaching was combined with 3D printing at a high temperature (120 °C). These scaffolds were then used to investigate the influence of these two parameters (porosity and stiffness) on the *in vitro* proliferation and osteogenic differentiation of hBMSC as well as maturation/activation of human monocyte-derived dendritic cells (Mo-DC).

## Materials and Methods

### PCL-NaCl Ink Preparation

PCL pellets with high molecular weight (Mn: 80 kDa) were used for NIPS-based low-temperature (LT) printing, while lower molecular weight pellets (Mn: 45 kDa) were used for high-temperature (HT) printing. To prepare 30% (w/v) PCL inks, PCL pellets (Sigma-Aldrich/Merck, USA) were fully dissolved in acetone (10 mL/gram PCL) at 40 °C for 2 h. Then, predetermined amounts of sieved NaCl particles (40 to 90 μm, Sigma-Aldrich/Merck) of 0%, 23%, 46%, and 92% (w/w) were added to the mixture to create PCL-NaCl composite inks.

### 3D Printing of PCL Inks

All inks were 3D printed by using a 3D-Bioplotter (EnvisionTech, Germany). For NIPS-based LT printing, PCL-NaCl solutions were placed into 30 cm^3^ plastic cartridges and loaded into the 3D Bioplotter. Stainless-steel nozzles (inner diameter of 500 μm) were utilized to extrude the inks at an average speed of 5 mm/s and an air pressure of 1.5–2.5 bar at 30 °C. The NIPS-based LT printing was performed in an absolute ethanol (EtOH) bath at room temperature (RT). Square-shaped scaffolds (15 × 15 mm) with four layers (layer height/slicing: 320 μm) were printed with a periodic internal pattern of 0/90°. The distance between the filaments was 1.5 mm (center-to-center distance). For the *in vitro* biological assessments, the distance between the filaments was 1.2 mm, and a shift of 0.6 mm was introduced in the *X*–*Y* plane for every third and fourth layer to enhance cell seeding efficiency. For HT printing, PCL-acetone solutions with varying NaCl concentrations were cast into glass Petri dishes filled with absolute ethanol and air-dried. The dried sheets were then cut into pellets using scissors. The pellets were additionally dried in a vacuum furnace at 30 °C overnight. Next, pellets were melted at 120 °C in a metallic cartridge of the 3D-Bioplotter and extruded through a nozzle of 0.6 mm diameter with an air pressure of 8 bar and an average printing speed of 10 mm/s. To remove the NaCl and create the pores, scaffolds were immersed in a 70% ethanol–water bath for 4 h before they were washed in deionized (DI) water for 5 days under stirring.

### Structural Analysis by Micro-Computational Tomography (micro-CT)

Micro computed tomography (micro-CT/μ-CT, Bruker SkyScan 1172, Belgium) was used to first assess NaCl particle size distribution, NaCl distribution within unwashed scaffolds, and scaffold porosity (micro porosity, total porosity, and pore size distribution). To visualize NaCl-based porosity, lower resolution (2k) micro-CT scans were conducted with 10 μm magnification. For NIPS-based porosity, pore size distribution and structure separation calculations were executed at 4k resolution with 1 μm magnification (pixel size). Micro-CT scanning parameters at different resolutions are presented in Table 1.

**Table 1:** Micro-CT Scanning Parameters at Different Resolutions

**Type of Scanning**	**Camera Pixel (μm)**	**Resolution Pixels**	**Pixel Size (μm)**	**Voltage (kV)**	**Exposure Time (ms)**	**Rotation Step**
**2k**	8.75	2000 × 1336	10	35	211	0.7
**4k**	8.75	4000 × 2672	1	35	1250	0.25

### Calculations of Total Microporosity in Filaments of LT and HT Scaffolds

Overall internal microporosity (i.e., Ø_μ_, via NIPS and salt leaching, if applicable) of LT and HT scaffold filaments were evaluated by weighing three scaffolds in wet and dry conditions. The amount of water absorbed by the scaffold was determined by subtracting the scaffold’s dry weight from its wet weight and hence gives microporosity within scaffolds’ all filaments in LT (E1). Advanced dual porosity in LT was calculated from equations below that it was introduced for the first time (E2). Whereas internal porosity induced by salt leaching in HT can be found via the ratio of NaCl leached to scaffold volume (E3). Detailed calculations can be found in the Supporting Information.
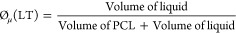

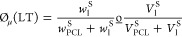
1
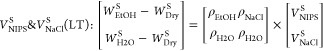
2
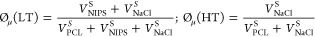
3

### Structural Characterization by Scanning Electron Microscopy (SEM)

To visualize the introduced microporosity, cross sections of scaffolds were produced by cutting scaffolds into liquid nitrogen by using pliers. Next scaffolds were coated with a layer of gold–palladium using a sputter coater (DSR1, Vac Techniche, UK). Scanning Electron Microscopy (SEM; Zeiss Leo Supra VP 55, Jena, Germany) was used to image samples at an acceleration voltage of 5 kV and 5–7 mm working distance.

### Mechanical Tensile Testing

Tensile stress tests were performed to assess the mechanical properties of 3D printed dog bone specimens (*N* = 3) with dimensions of 0.3 mm thickness (1 layer), 5 mm width, and 10 mm gauge length. Uniaxial tensile load was applied to the printing direction. The tests were conducted by using a universal testing machine (MTS, 858 mini Bionix II instrument, Eden Prairie, MN, USA) at a strain rate of 0.1 mm/s.

### Accelerated Degradation

The degradation was calculated by measuring weight loss after 14 days in a 1 M NaOH aqueous solution at RT as shown in E4. Degraded samples were imaged with SEM to evaluate the morphological changes of the samples.
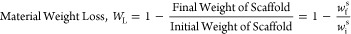
4

### Scaffold-hBMSC Interactions

#### hBMSC Culture

The cytocompatibility and osteogenic performance of the printed scaffolds were evaluated with hBMSC that were isolated and characterized under ethical approval from the Regional Committee for Medical and Health Research Ethics in Norway (2020/7199/REK sør-øst C).^20^ Cells were expanded in growth medium (Alpha-Minimum Essential Medium, α-MEM, Gibco, ThermoFisher Scientific, MA, USA) supplemented with 10% fetal bovine serum (FBS, HyClone, GE Healthcare, Utah, USA) and 1% antibiotics (100 U/ml penicillin and 0.1 mg/mL streptomycin Gibco, ThermoFisher Scientific) at 37 °C and 5% CO_2_. For osteogenic differentiation, the growth medium was supplemented with 10 nM dexamethasone, 0.05 mM l-ascorbic acid, and 10 mM β-glycerophosphate before addition on scaffolds. In all experiments, cells from passages 2–4 were used, and the medium was replaced twice a week.

#### Viability and Proliferation of hBMSC Seeded on Scaffolds

To ensure proper fitting within 48-well low adherent plates (Sarstedt, Numbrecht, Germany), disc-shaped scaffolds (Ø = 8 mm) were punched out of the printed squares. The punched scaffolds were disinfected using 70% ethyl alcohol for 1 h before UV sterilization for 30 min. Sterilized scaffolds (HT0, HT92, LT0, and LT92) were prewetted overnight in growth medium before seeding cells at a density of 1 × 10^5^ cells/scaffold in 50 μL cell suspension. After 1.5 h of initial incubation, wells were supplemented with 750 μL of media.

To assess the cell viability, live/dead (ThermoFisher Scientific) staining was employed. After washing with PBS (Dulbecco’s Phosphate-Buffered Saline, Gibco, ThermoFisher Scientific, USA), samples were incubated for 40 min at RT in the dark in a PBS solution containing EthD-1 and Calcein-AM. Subsequently, the scaffolds were washed with PBS and imaged using a fluorescence microscope (Nikon, Eclipse 80i, Tokyo, Japan).

Cell metabolic activity based on the PrestoBlue (PB) assay was employed to evaluate viability of hBMSC on scaffolds. On days 1, 7 and 14, cell-seeded scaffolds were transferred to a new 48-well plate and fresh medium with 10% (v/v) ready-to-use PrestoBlue solution (Invitrogen, ThermoFisher Scientific, MA, USA) was added to each well, followed by a 15 min incubation at 37 °C. The fluorescence (550–590 nm) was then measured using a microplate reader (VarioskanTM LUX, ThermoFisher Scientific).

For proliferation assessment, the Quant-iTTM PicoGreen DNA kit (Invitrogen, ThermoFisher Scientific, MA, USA) was utilized. On days 1, 7, and 14, scaffolds were washed with PBS, treated with 0.1% Triton-X/PBS, and stored at −80 °C. After two freezing-thawing cycles and 40 s of sonication, 50 μL of each sample and an equal amount of working PicoGreen solution were combined in a 96-well according to the manufacturer’s protocol. Fluorescence at 480/520 nm was measured using a microplate reader.

#### Alkaline Phosphatase Activity

Alkaline phosphatase activity (ALP) was analyzed from the same Triton- X 100 lysates used for the proliferation test using a commercial kit (p-Nitrophenyl Phosphate Liquid Substrate System, Sigma-Aldrich/Merck). Briefly, equal volumes of ALP working solutions and samples (50 μL) were pipetted into the wells of a 96-well plate and incubated for 30 min at 37 °C. Absorbance at 405 nm was measured using the microplate reader.

#### Alizarin Red S Staining

On day 28 of culture in osteogenic medium, Alizarin red S staining (Sigma-Aldrich) was used to evaluate the osteogenic differentiation of the hBMSC by detecting calcium deposition. The cells were fixed with 4% paraformaldehyde for 15 min and then stained with 2% Alizarin red solution (pH 4) for 30 min at RT. After several washes with milli-Q water, the scaffolds were air-dried and imaged using a stereomicroscope (Leica M205C, Wetzlar, Germany). To quantify the staining, samples were incubated in 1 mL of 100 mM cetylpyridinium chloride (Sigma-Aldrich/Merck), and the absorbance was measured at 540 nm. Scaffolds without cells were stained to serve as the controls.

#### Immunofluorescence Staining

Seeded scaffolds were fixed in 4% paraformaldehyde (PFA) for 10 min at RT and then permeabilized with 0.1% Triton X-100 in PBS for 10 min at RT. To block nonspecific binding, 10% normal goat serum (NGS) (ab7481, Abcam, UK) with 1% Bovine Serum Albumin (BSA) (Sigma-Aldrich/Merck) in PBS was applied for 2 h at RT. Subsequently, the samples were incubated overnight at 4 °C with RUNX2 rabbit polyclonal antibody (Product # PA5-82787, Invitrogen) at 1:200 dilution or Collagen I recombinant rabbit monoclonal antibody (ST58-04) (Product # MA5-32178) at 1:200 dilution in diluted blocking solution (1% NGS with 1% BSA in PBS). Following primary antibody incubation, Goat anti-Rabbit IgG secondary antibody (Alexa Fluor 647, Invitrogen, ThermoFisher Scientific, MA, USA) was applied at 1:500 dilution in the blocking solution for 1 h at RT to label the primary antibodies. Filamentous actin (F-actin) and nuclei were counterstained with Phalloidin Alexa Fluor 488 (1:250; A12379, ThermoFisher Scientific) and 4′,6-diamidino-2-phenylindole (DAPI, 1:5000; 62247, ThermoFisher Scientific) for 1 h at RT. After staining, the samples were washed five times for 5 min each with PBS. Imaging was performed using an Andor Dragonfly 5050 high-speed confocal microscope and Fusion software (Oxford Instruments, Abingdon, UK). Images were acquired with a resolution of 1024 × 1024 by using a high-speed iXon 888 Life EMCCD camera.

### Scaffold-Dendritic Cell Interactions

#### Monocyte Isolation and Monocyte-Derived Dendritic Cell Culture

Buffy coat preparations were obtained from healthy donors from the Bergen Blood Bank, Haukeland University Hospital, Bergen (AIT-70729). Peripheral blood mononuclear cells (PBMC) were isolated using Ficoll density gradient centrifugation, and subsequently CD14^+^CD16^low^ monocytes were isolated by magnetically activated cell sorting (MACS) using Classical Monocyte Isolation Kit following the manufacturer’s instructions (Miltenyi, Bergisch Gladbach, Germany).

Scaffolds were prewet with complete medium made from RPMI medium containing human 5% AB serum (Sigma-Aldrich/Merck) and 1% antibiotics, for a minimum of 1 h before cell seeding. Unlabeled monocytes were seeded in complete medium at cell density of 2.5 × 10^5^ cells/cm^2^ in standard 12-well tissue culture plates (TCP, 2D control) and 8.75 × 10^5^ cells/scaffold (PCL-HT92 and PCL-LT92). To induce differentiation into dendritic cells after 1–2 h of seeding, medium was supplemented with 100 ng/mL granulocyte-macrophage colony-stimulating factor (GM-CSF; R&D, USA) and 20 ng/mL IL-4 (Miltenyi, Germany) and cultured for 7 days. Half the medium was replenished every 3 days with cytokine-supplemented medium.

#### Dendritic Cell Morphology and Activation

The attachment and spreading of monocyte-derived dendritic cells (Mo-DC) after 6 days postseeding on scaffolds or tissue culture plate (2D TCP) was evaluated by SEM (Phenom XL G2, desktop SEM) at an acceleration voltage of 10 kV.

To evaluate the differentiation and activation state of the DC culture on scaffolds, cells were collected from 2D TCP and scaffolds. Unspecific binding was blocked with FcR block (Miltenyi, Germany) for 10 min at 4 °C. Cells were stained with viability dye, APC-eFluor780 anti-CD40, PE anti-CD11c, FITC anti-HLA-DR (Thermo Fisher), APC anti-CD14, FITC anti-CD16 and APC anti-CD80 (BioLegend) at dilution of 1:200 for 30 min at 4 °C. The stained samples and their unstained controls were acquired on a BD Accuri C6 flow cytometer. Dead cells and doublets were excluded from the analysis. Data was analyzed using flow cytometry software (FlowJoV10.6. 2, LLC, Ashland, USA).

Differentiation of DC was also evaluated at the gene level using the real time quantitative polymerase chain reaction (qPCR). Total RNA was collected at day 7 using a Qiagen Mini RNeasy kit (Qiagen, Germany). For cDNA synthesis, the High-Capacity cDNA Reverse Transcription Kit (Applied Biosystems, CA, USA) was used following the manufacturer’s protocol. Taqman gene expression (Applied Biosystems, USA) assays were used to detect mRNA levels of immunomodulatory human genes in DC, including *TGFB* (Hs0098133_m1), *IL10* (Hs00961622_m1), *IL1B* (Hs01555410_m1) and *TNF* (Hs00174128_m1). Data were analyzed using the comparative C_T_ method with glyceraldehyde-3-phosphate dehydrogenase (*GAPDH*, Hs99999905_m1) as an endogenous control. DC on 2D TCP served as reference samples.

#### Statistical Analysis

Statistical analysis was conducted by using One-way ANOVA with Tukey’s multiple comparison test or Student’s two-tailed *t* test in GraphPad (version 5, California, USA). Data are expressed as the mean ± the standard deviation (SD). Differences were considered statistically significant at *p* < 0.05.

## Results

### PCL Inks, Porosity Design, Printing, and Postprinting Washing

**Figure 1:**
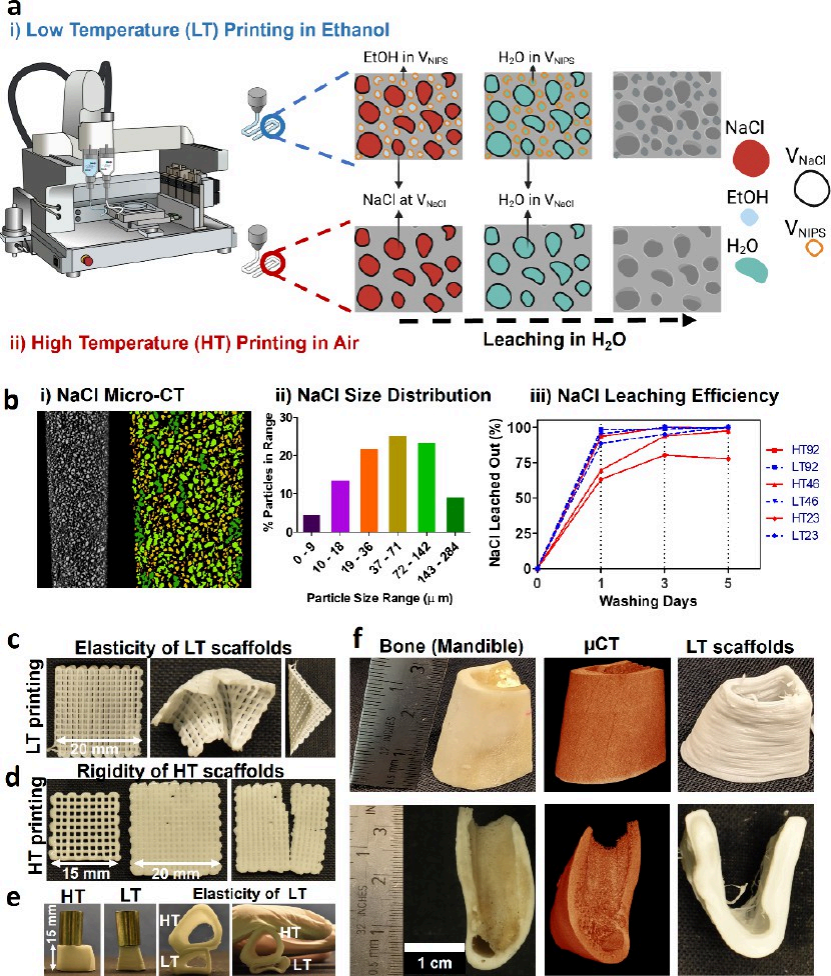
(a) Schematic illustration of low temperature (LT) and high temperature (HT)-based 3D printing combined with salt (NaCl) leaching and/or nonsolvent-induced phase separation (NIPS). (b) NaCl particle size distribution obtained by micro-CT and washing/leaching efficiency of LT and HT scaffolds (*N* = 3). (c) Flexibility and foldability of NIPS-based LT scaffolds. (d) Stiffness and brittleness of the HT scaffolds. (e) Mechanical stability of the 3D printed structures under longitudinal compressive loading by metal weights (40 g) and their flexibility under axial compressive loading by hand. (f) Printability and buildability of NIPS-based LT printing technique showing printing of real size sheep mandibular bones from micro-CT images.

Inks were synthesized by dissolving PCL in acetone with or without NaCl particles. The inks were then successfully printed either at high temperature (HT = 120 °C) in air or at low temperature (LT = 30 °C) in ethanol (Figure 1a). By introducing the distance between the printed filaments as a design parameter of 3D printing, bulk macroporosity (open channels) was created. By including NaCl particles in the inks and leaching them out after printing, internal large microporosity was introduced in the printed filaments. The LT-based printing in ethanol bath resulted in the introduction of high levels of small microporosity in the printed filaments. Although NaCl particles were sieved between 40 and 90 μm, the micro-CT analysis showed wider size range with around 60% of the particles between 19 and 142 μm (Figure 1b). The efficient removal of these particles in postprinting by washing in water was concentration dependent: the higher the initial salt concentration within the ink, the faster and more complete the salt leaching as shown in Figure 1b_**iii**_. The leaching of NaCl from LT-based scaffolds was faster than that from their HT counterparts. Importantly, more than 90% of NaCl leached out of LT92 and HT92 after 1 day of washing. Unlike LT23 which demonstrated 94% salt removal after 3 days, HT23 displayed only 77% removal after 5 days. All LT-based scaffolds were completely leached at the end of day 5 regardless of the initial NaCl content.

Figure 1c-e shows the effect of the printing temperature on the printed structure’s mechanical properties. LT-based printing in ethanol resulted in flexible scaffolds that can be bent. The HT-based printed structures were brittle and were found to break on bending. While both LT and HT scaffolds were mechanically stable under compressive longitudinal load (40 g), only LT scaffolds demonstrated high flexibility under axial compressive loading by hand (Figure 1e). Compared to the conventional HT-based printing of PCL, which is known to produce high shape fidelity, the LT NIPS-based printing technique demonstrated excellent printability and buildability that allowed for the fabrication of real size sheep mandibular bone-like structures (3 cm high) from micro-CT images (Figure 1f and Figure S2a). Moreover, LT scaffolds showed good dimensional stability with reversible slight shrinkage after printing in ethanol, washing in water, drying in air, and wetting again (supplementary Figures S2–S4).

### Salt Leaching-Based Printing Induces Large Micropores

**Figure 2:**
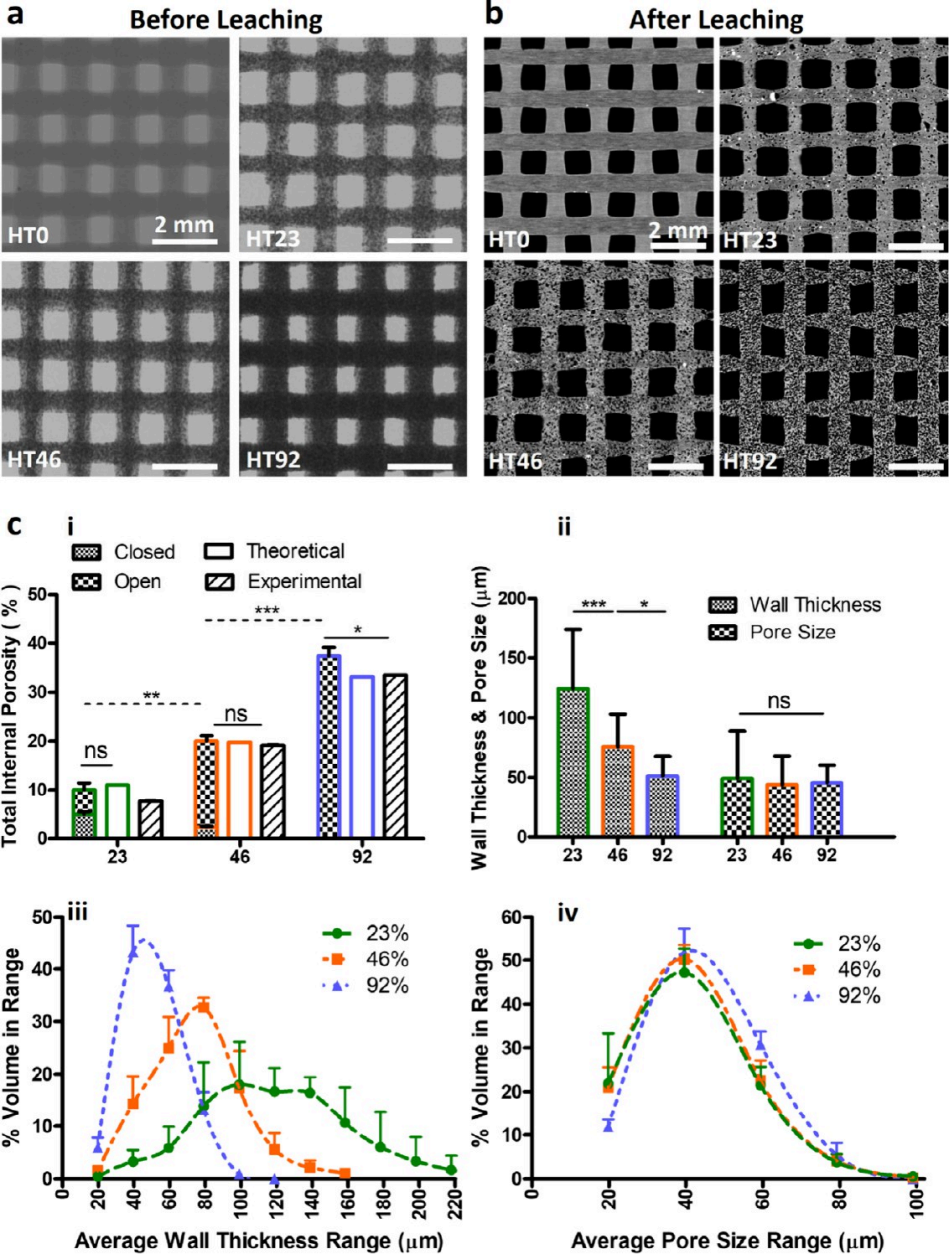
Micro-CT structural analysis of HT scaffolds with various content of NaCl (0, 23, 46 and 92% w/w) (a) before and (b) after leaching. (c) Quantification analysis of pores induced by NaCl leaching; (i) total internal porosity in comparison with theoretical and experimental calculated values, (ii) pore wall thickness and size obtained from distribution plots (iii, (iv). *N* = 3. *p < 0.05, **p < 0.01, ***p < 0.001.

**Figure 3:**
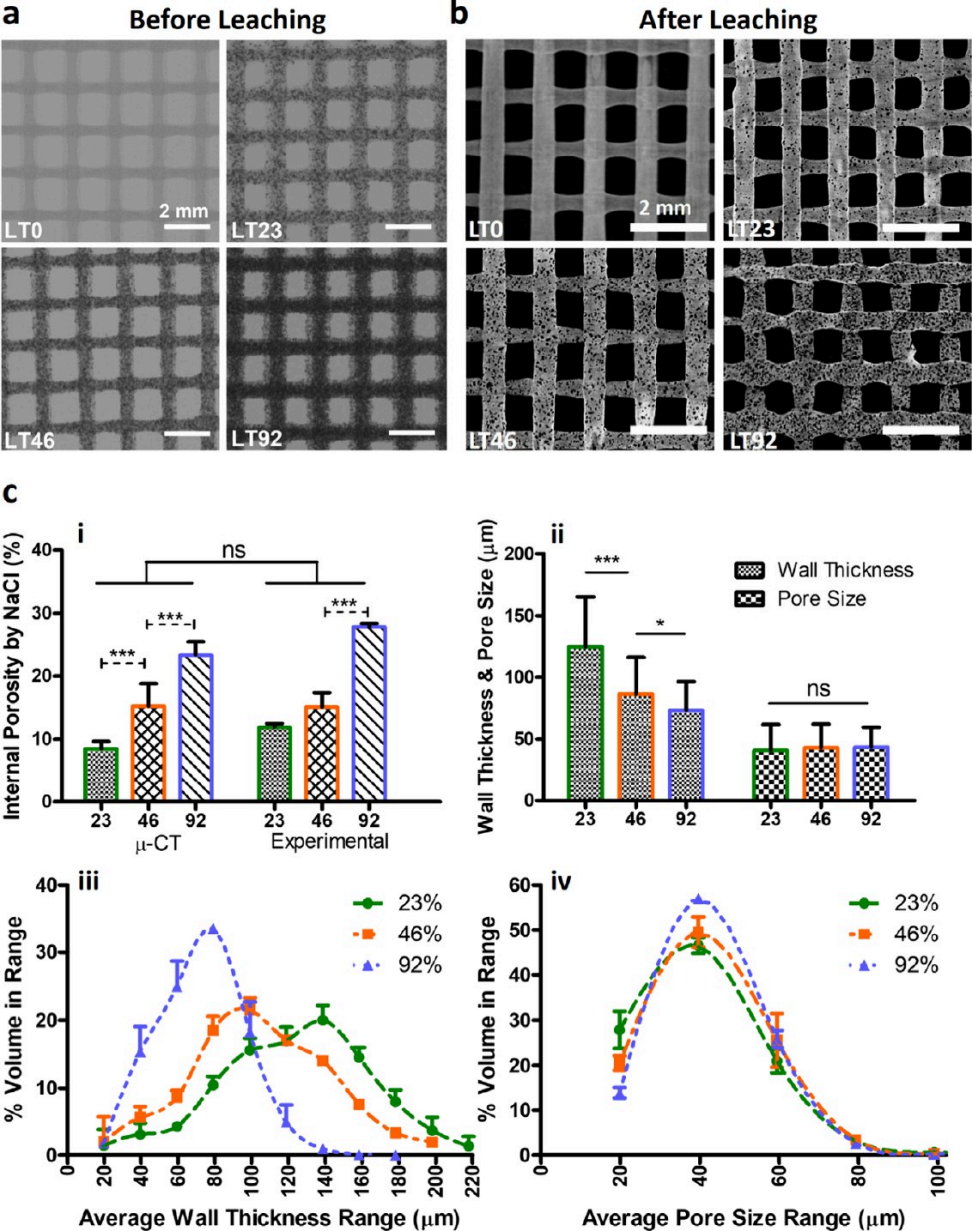
Micro-CT structural analysis of LT scaffolds with various content of NaCl (0, 23, 46 and 92% w/w) (a) before and (b) after leaching. (c) Quantification analysis of pores induced by NaCl leaching; (i) total internal porosity in comparison with experimental calculated values, (ii) pore wall thickness and size obtained from distribution plots (iii, iv). *N* = 3. *p < 0.05, **p < 0.01, ***p < 0.001.

The filaments’ internal porosity was visualized and quantitatively analyzed by micro-CT before and after salt leaching in water, as shown in Figure 2 and 3. The micro-CT images confirmed the high printing quality and shape fidelity of LT-based printing which was close to the quality of HT-based printing. Furthermore, the images showed excellent distribution of the NaCl particles and porosity throughout the printed scaffolds before and after leaching. Furthermore, the porosity, black voids surrounded by the polymer matrix, increased with salt concentration. This is quantitatively presented in Figure 2c_**i**_ and 3c_**i**_. HT92 and LT92 scaffolds demonstrated the highest levels of internal microporosity compared to HT23 and LT23.

The expected total microporosity resulting from salt leaching in LT- and HT-based printing was theoretically calculated as 0%, 11%, 19.8%, and 33.1% for salt concentrations of 0, 23, 46, and 92% w/w, respectively. This calculation was based on the volume-weight-density relationship for NaCl. The total actual microporosity (measured experimentally) of HT scaffolds was found to be 0%, 7.7%, 19.2%, and 33.5%. The total microporosity results obtained from the micro-CT was 10%, 20% and 37.4% for HT23, HT46 and HT92 respectively. Regarding LT scaffolds, the total actual microporosity (measured experimentally) was found to be 0%, 11%, 15%, and 27% whereas the total microporosity results obtained from the micro-CT was 8.5%, 12.6%, and 23.3% for LT23, LT46 and LT92, respectively. More importantly, low salt concentrations in HT-based printing resulted in closed porosity (in HT23) due to poor water diffusion and suboptimal removal of the salt. Around 50% (5% ± 0.5) of the total salt-induced porosity in HT23 scaffolds comprised closed pores, whereas this ratio was around 10% for HT46. Conversely, all pores in HT92 were found to be open and well interconnected via micro-CT (Figure 2c_**i**_). Similarly, all pores in LT-based printed scaffolds were observed to be completely open in porosity, regardless of initial NaCl content, due to the high level of NIPS porosity.

In addition to the total porosity, pore size and pore wall thickness were analyzed and presented in Figure 2c_**ii**_**-**_**iv**_ and 3c_**ii-iv**_. In contrast to the increased total porosity with salt concentration, the average pore wall thickness decreased with increasing NaCl content. Decreasing wall thickness from 124.4 ± 50 μm in HT23 group to 51.5 ± 16 μm in HT92 group resulted in the formation of interconnected pores. Yet, this decrease in pore wall thickness did not affect the average pore size. The pore size was dependent on the size of the NaCl, and all scaffolds exhibited a unimodal pore size distribution ranging from 20 to 100 μm with a single peak around 40 μm (Figure 2c_**ii-iv**_). Similarly, the calculated average wall thickness values for LT23, LT46, and LT92 scaffolds were found to be 125 ± 41, 105 ± 86, and 73.3 ± 23 μm (Figure 3c_**ii-iv**_).

### NIPS-Based Printing Induces a High Level of Interconnected Small Microporosity

**Figure 4:**
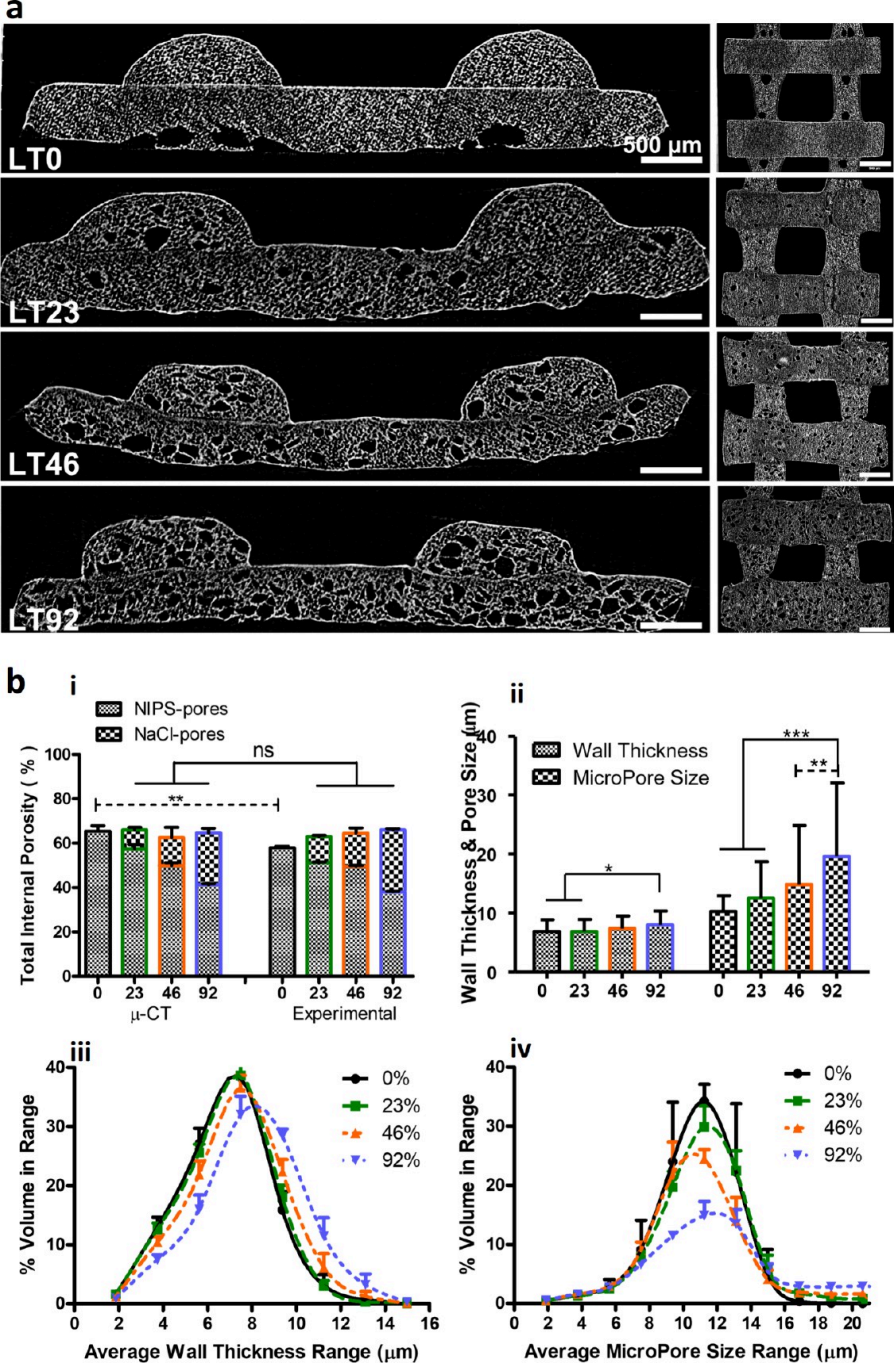
(a) High resolution (4k) micro-CT analysis of NIPS-based LT scaffolds with various contents of NaCl (0, 23, 46 and 92% w/w) after leaching. (b) Quantification analysis of micropores induced by both NIPS and NaCl leaching. (i) Total internal porosity by μCT in comparison with experimentally calculated values, (ii) pore wall thickness and pore size obtained from distribution plots (iii, iv). *N* = 3. *p < 0.05, **p < 0.01, ***p < 0.001.

High resolution micro-CT scanning was employed to visualize and quantify microporosity induced by NIPS-based printing at a low temperature (Figure 4a,b). PCL scaffolds (LT0) without salt leaching porosity exhibited total interconnected porosity of 65% ± 2.5 with more uniform pore wall thickness and size. LT0 exhibited a unimodal pore wall thickness distribution ranging from 2 to 15 μm with a single peak around 7 μm, while pore size had a distribution range from 2 to 17 μm and single peak around 10 μm. Although the addition of NaCl resulted in a gradual decrease in the total percentage of these small microporosities, the total porosity (salt leach and NIPS) did not change significantly and remained around 60% (Figure 4b_**i-ii**_). The pore and the wall thickness distribution curves exhibited a tendency to shift toward the right, suggesting an enlargement in pore size and thin wall thickness due to the increased NaCl content (Figure 4b_**iii-iv**_). Unlike the thin pore wall thickness in scaffolds with lower NaCl content (LT23 and LT46), HT92 demonstrated a significant increase in wall thickness (*p* < 0.05). Furthermore, the average micropore size notably increased (*p* < 0.01) with higher salt content, particularly evident in HT46 and HT92.

### Confirmation of Multiscale Porosity of the Printed Scaffolds by SEM

**Figure 5:**
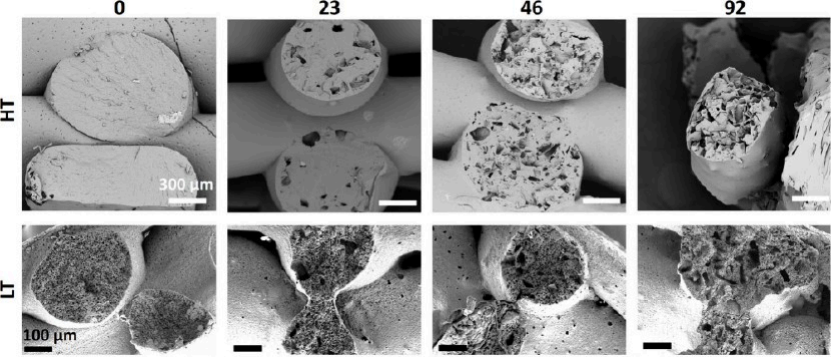
SEM structural analysis of cross sections of 3D printed HT and LT scaffolds with various contents of NaCl (0, 23, 46 and 92% w/w) after leaching.

Scanning electron micrographs of scaffolds after washing are presented in Figure 5. Cross sections of the printed filaments demonstrated that the induced internal porosity varied depending on the printing temperature and concentration of NaCl. HT-based printing of PCL in air without addition of NaCl resulted in solid nonporous filaments. NIPS-based LT printing in ethanol produced filaments with high levels of internal small microporosity. Addition of NaCl to the PCL inks and leaching it after printing induced internal microporosity with a pore size larger than NIPS-based pores. Altogether, SEM images further validated the previously observed and quantified results from the micro-CT.

### Induced Microporosity Significantly Affects Mechanical Property and Degradation Rates

**Figure 6:**
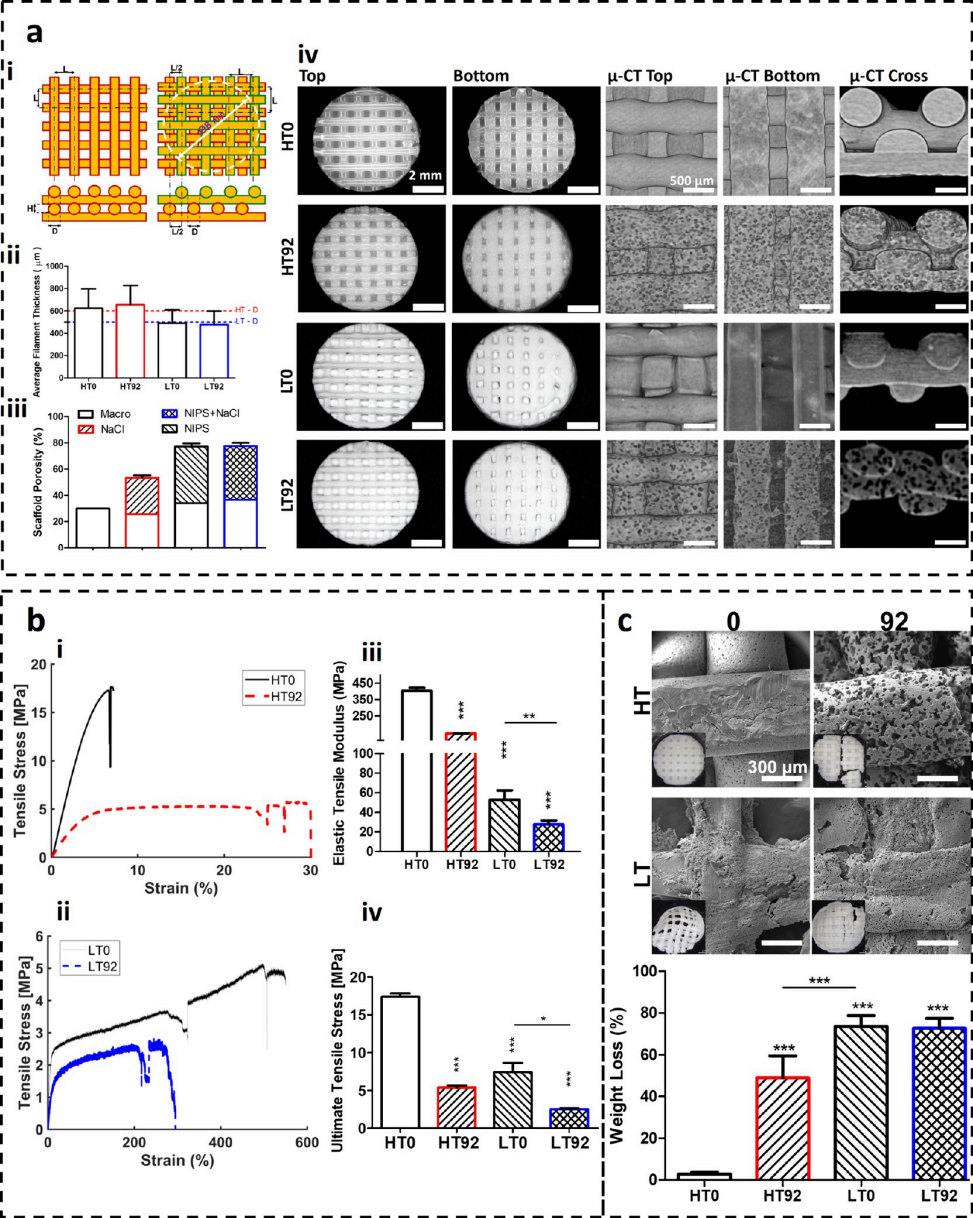
(a) 3D printing and characterization of selected LT and HT scaffolds with porosity produced by leaching out 0% and 92% NaCl. (i) Theoretical design of a grid pattern (0–90°) with and without *X*–*Y* shift. Center-to-center distance between filaments, *L* = 1.2 mm; layer height, *H* = 0.32 mm; filament diameter of HT and LT, *D* = 0.6 mm, and 0.5 mm, respectively. (ii) Average filament thickness, (iii) overall scaffold macro (channel) and micro (NIPS/NaCl) porosity, and (iv) structural characterization by stereomicroscopy and micro-CT images showing different views and sections. (b) Mechanical tensile test of dog-bone specimens. Stress–strain curves for HT (i) and LT (ii) scaffolds. Elastic modulus (iii) and ultimate tensile stress (iv) of printed scaffolds (*N* = 3). (c) *In vitro* accelerated degradation study and SEM images of the scaffolds on day 14 (*N* = 3). Asterisks indicate significant statistical differences between control group (HT0) and other groups, while underlined asterisks indicate that of between two test groups. *p < 0.05, **p < 0.01, and ***p < 0.001.

Based on the previous microstructural analysis, highly porous groups (HT92 and LT92) and their control counterpart groups (HT0 and LT0) were selected for further mechanical characterization, degradation analysis, and cell-scaffold interaction studies (Figure 6a). For cell culture, an X-Y layer shift in the grid pattern (0–90°) was introduced to support cell seeding efficiency. The micro-CT and stereomicroscopy showed the shifted layers in all groups. Additionally, the NIPS-based LT printing slightly decreased the filament thickness.

Mechanically, the printing modification by salt leaching and NIPS significantly changed the mechanical properties of the conventionally printed PCL at high temperature (HT0) as shown in Figure 6b. NIPS-based LT specimens exhibited high ductility due to NIPS-based microporosity, resulting in significantly larger strain compared with the brittle and stiff behavior of the HT specimens. The stress–strain curves also provided insights into the relation between toughness and NaCl concentrations in the different specimens: the toughness in HT printing increased, while in LT printing it decreased with higher NaCl-induced porosity. In HT groups, the increase in porosity due to the higher salt content resulted in greater elongation and reduced tensile strength. Furthermore, introduction of internal microporosity resulted in a significant reduction in the modulus of elasticity and tensile strength of the printed samples.

Since PCL is known to have a slow degradation rate, an accelerated degradation test in NaOH 0.1 M aqueous solution was performed for up to 14 days (Figure 6c). The calculated change in the mass over time indicated that the NIPS-based porosity in LT0 and LT92 groups significantly enhanced the degradation compared to that in the nonporous filament control group (HT0). Similarly, the introduction of NaCl-based porosity in HT92 significantly reduced the weight of scaffolds compared to the HT0 group. However, NaCl-based porosity did not change the degradation profile of LT92 compared to LT0. A summary of the properties of selected scaffolds is presented in Table 2.

**Table 2:** Summary of the Properties of the Selected Scaffolds

	**Total porosity (%)**	**Modulus of elasticity (E) (MPa)**	**Strain (%)**	**Tensile strength (MPa)**	**Accelerated weight loss (%)**
**HT0**	30.1 ± 2.16	403 ± 19	6.6 ± 0.1	17.4 ± 0.4	3 ± 0.75
**HT92**	53.5 ± 1.80	143 ± 2	21.5 ± 2.2	5.4 ± 0.3	49 ± 10.50
**LT0**	77.2 ± 2.44	53 ± 10	435 ± 105	7.4 ± 1.4	73.5 ± 5.10
**LT92**	77.6 ± 2.50	28 ± 4	220 ± 28	2.6 ± 0.1	72.7 ± 4.70

### Effect of Porosity and Stiffness on hBMSC Viability and Proliferation

**Figure 7:**
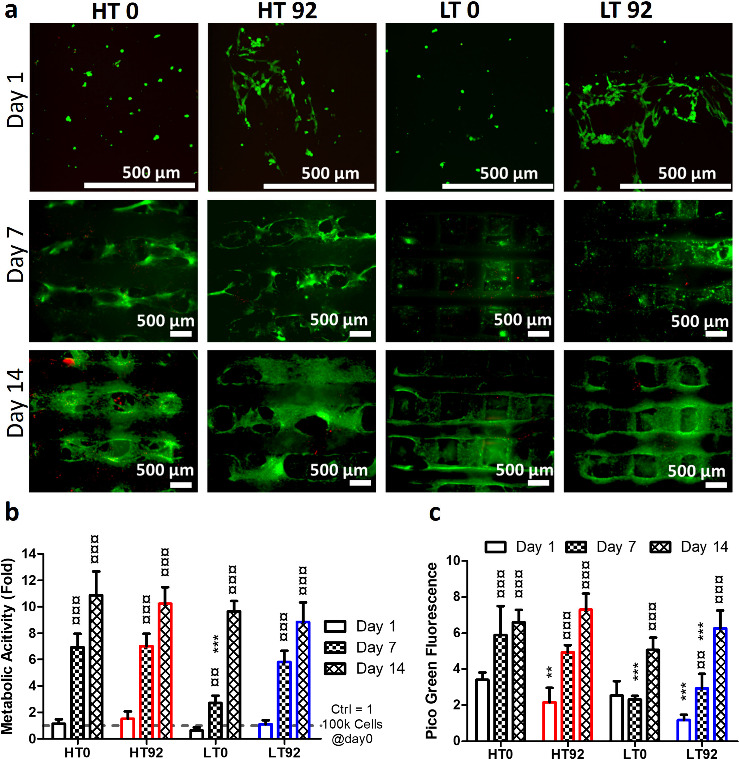
*In vitro* cytocompatibility of hBMSC cultured on LT and HT scaffolds with porosity produced by leaching out 0% and 92% NaCl. (a) Fluorescence images of live and dead cytotoxicity showing live cells (green) and dead cells (red). (b) Metabolic activity by the PrestoBlue assay (*N* = 6). (c) Cell proliferation by the PicoGreen dsDNA assay (*N* = 6). Asterisks (*) indicate significant statistical differences between control group (HT0) and other groups at the same time point, while currency signs (¤) indicate the difference between time points of the same group. *p < 0.05, **p < 0.01, ***p < 0.001, ^¤^*p* < 0.05, ^¤¤^*p* < 0.01, ^¤¤¤^*p* < 0.001.

At all-time points and regardless of the salt content and hence porosity, all 3D printed scaffolds displayed no signs of cytotoxicity, as characterized by live–dead staining (Figure S5, S6 and Figure 7a). Cells maintained viability, with most cells living (green) and few dead cells (red), indicating good cytocompatibility of the scaffolds. Morphologically, introduction of NaCl-based porosity (large micropores) in LT92 and HT92 groups enhanced cell attachment and morphology on day 1. Cells on HT0 and LT0 scaffolds displayed reduced spreading and rounded shape, while on HT92 and LT92 cells spread more with more elongated morphology. Over time, cells on all scaffold groups elongated, creating a uniformly dispersed network within the scaffold space. Interestingly, scaffolds with NaCl-induced porosity exhibited a more interconnected network of both intra- and interfilaments compared to those without NaCl-induced porosity (Figures S5–S6).

The absence of cytotoxic effects of pre- and postprinting treatments was confirmed by measurement of cell metabolic activity as shown in Figure 7b. The metabolic activity of the cells increased significantly from day 1 to day 14 in all groups. Moreover, there was no significant difference between scaffolds at any time points except for LT0, which was lower (*p* < 0.01) than HT0 control group on day 7. Furthermore, the results obtained from dsDNA quantification confirmed the ability of the printed scaffolds to support cell proliferation (Figure 7c). The number of cells increased significantly on all scaffolds from day 1 to day 14. However, the microporosity reduced cell proliferation compared to the HT0 group on days 1 and 7. On day 7, cells cultured on both LT scaffolds proliferated significantly less than the HT0 group. Nonetheless, these differences became insignificant on day 14, thus confirming the ability of the printed scaffold to maintain cell proliferation over time.

### Effect of Porosity and Stiffness on hBMSC Osteogenic Differentiation

**Figure 8:**
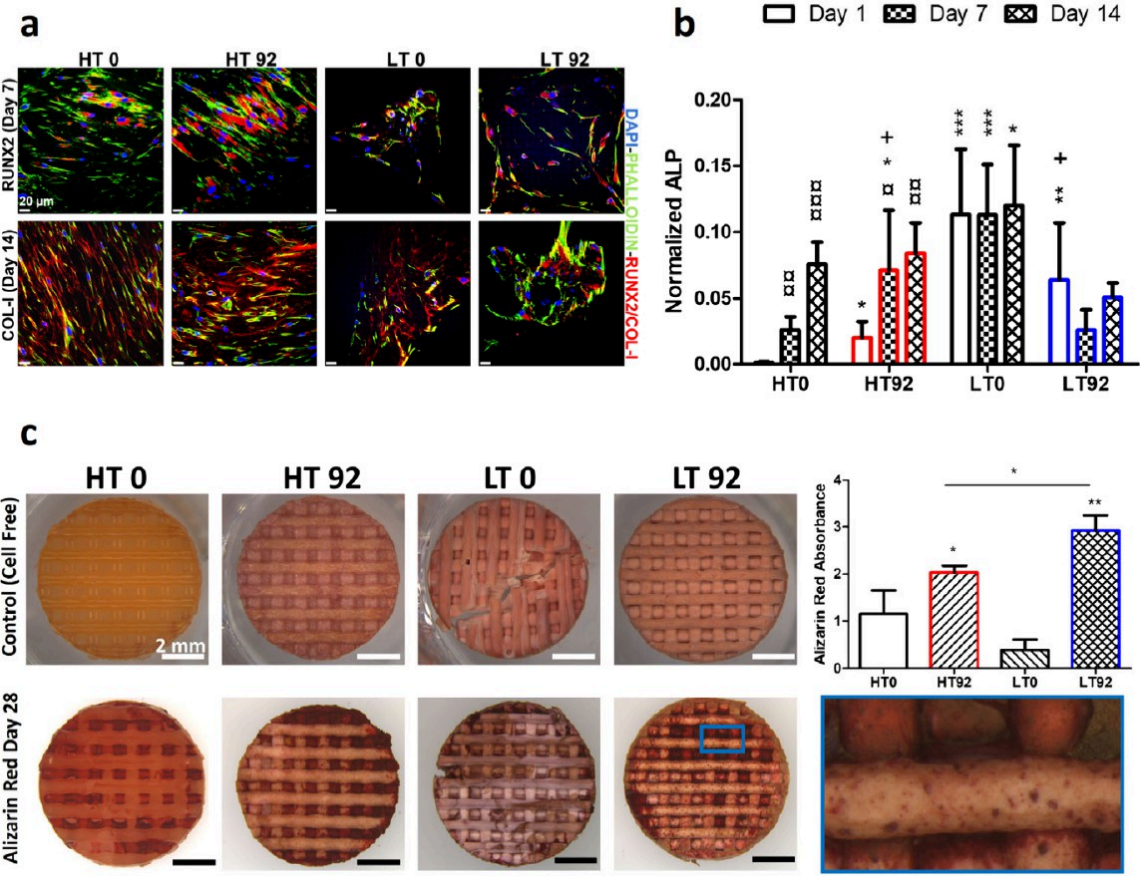
*In vitro* osteogenic differentiation of hBMSC cultured on LT and HT scaffolds with porosity produced by leaching out 0% and 92% NaCl. (a) Confocal microscopy fluorescent images of immunostained cellular components showing RUNX-2 (red) expression, Collagen I (red) production, F-actin filaments (green), and nuclei (blue) in cells cultured for 7 and 14 days in osteogenic medium, respectively. (b) Alkaline Phosphatase (ALP) activity normalized to dsDNA (*N* = 6). (c) Images showing mineralization by Alizarin Red S on day 28 and its quantification (*N* = 3). Asterisks (*) indicate significant statistical differences between the control group (HT0) and other groups at the same time point, while the currency sign (¤) indicates the difference between time points of the same group. The plus symbol (+) indicates differences between LT92 and HT92 at the same time point. *p < 0.05, **p < 0.01, ***p < 0.001, ^¤^*p* < 0.05, ^¤¤^*p* < 0.01, ^¤¤¤^*p* < 0.001 and ^*+*^*p* < 0.05.

To evaluate the effect of different porosity and stiffness on the osteogenic differentiation of cells, expressions of RUNX2 (osteogenic transcription factor) and Collagen I were analyzed by immunofluorescence, as shown in Figure 8a. Several cells in all groups were positively expressing RUNX2 and Collagen I, which confirmed the ability of scaffolds to support osteogenic differentiation of the cells. Although the immunofluorescence images provide only qualitative data, they show that the HT groups promoted greater cell organization and osteogenic marker production compared to the LT groups. ALP, an early marker of osteogenic differentiation, was also quantified after normalization to the total cell number (Figure 8b). ALP activity was found to be elevated in HT groups from day 1 to day 14, but not in LT groups. LT0 demonstrated the highest level of ALP compared with other groups. On day 1, NaCl-based and/or NIPS-based microporosity significantly increased ALP activity in HT92, LT0 and LT92 compared to HT0 (without internal filament porosity). However, NaCl-based porosity in LT92 decreased ALP compared to that in LT0 at all time points.

To investigate the late stages of osteogenic differentiation, cells cultured on scaffolds were stained with Alizarin red S on day 28 of culture to detect calcium deposition, as shown in Figure 8c. Compared to cell-free scaffolds, all scaffolds seeded with cells displayed substantial color change. Particularly, HT92 and LT92 scaffolds exhibited extensive mineral formation compared to the control groups. After quantification, LT0 demonstrated the lowest amount of mineralization. The porous salt-leached scaffolds demonstrated more mineralization compared to their counterpart groups with the highest level of mineralization found on LT92 scaffolds followed by HT92 group.

### Effect of Porosity and Stiffness on Morphology and Activation of Mo-DC

**Figure 9:**
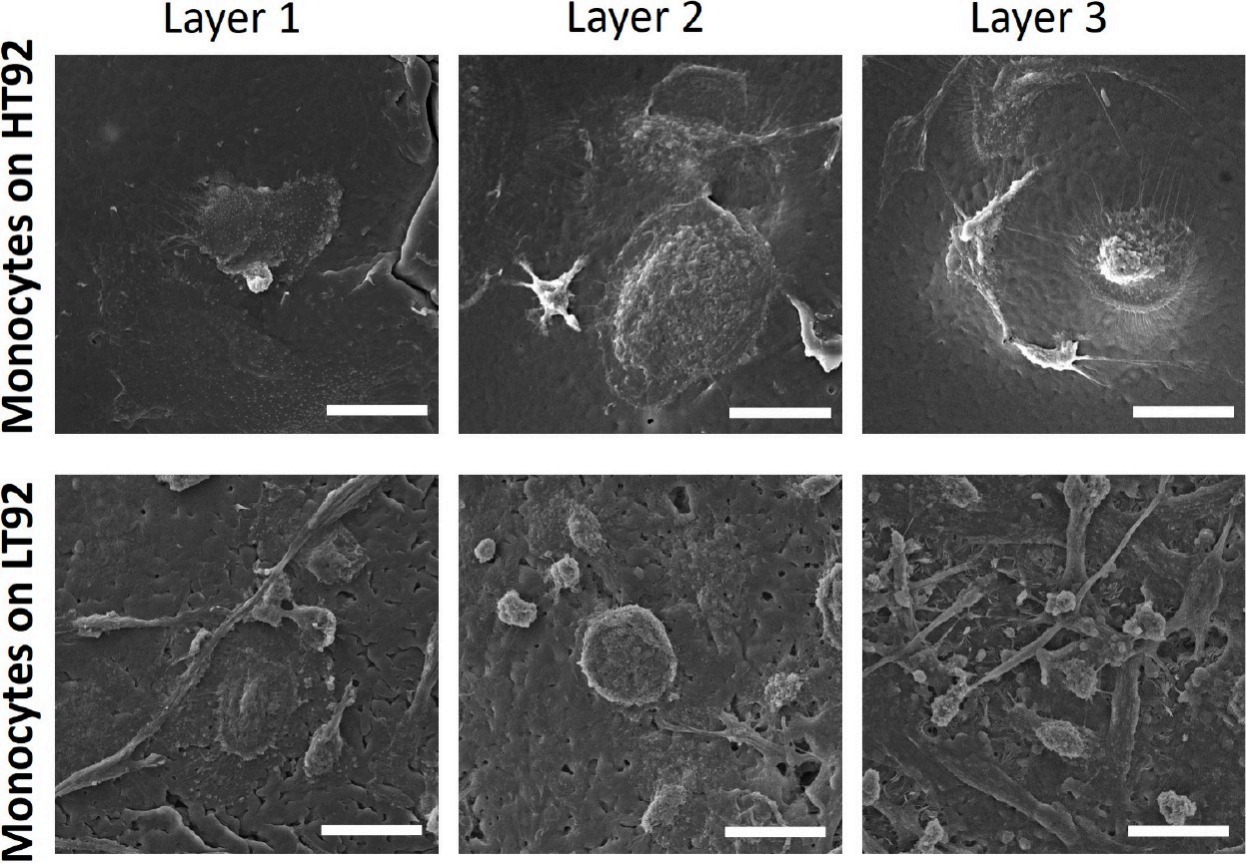
Scanning electron microscopy images showing undifferentiated classical monocytes (CD14^+^ CD16^low^) morphology on HT92 and LT92. Scale bar: 20 μm.

**Figure 10:**
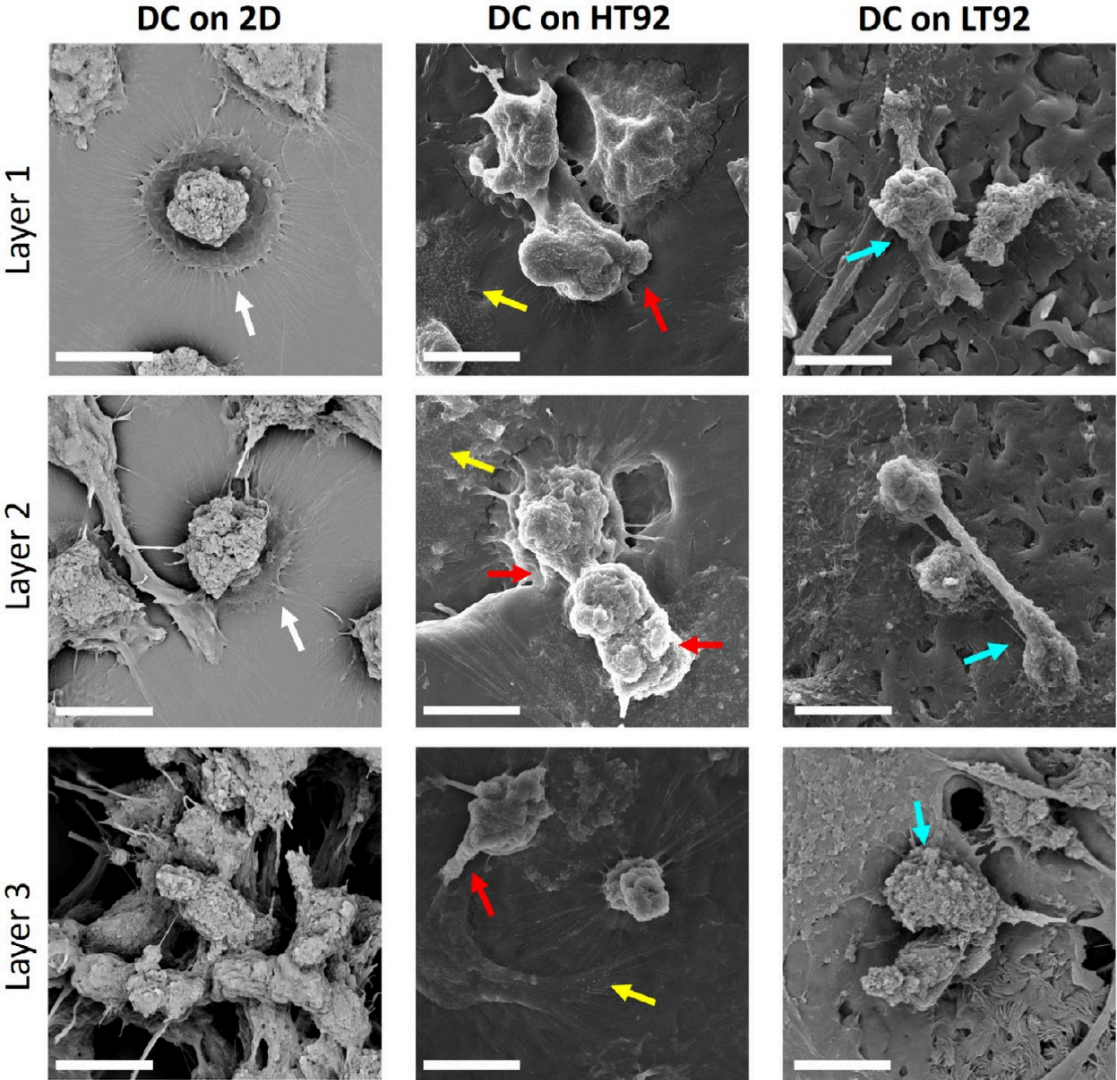
Scanning electron microscopy images showing Mo-DC morphology on HT92, LT92 and TCP samples. White arrows indicate needle-like structures and ruffles present within the cell body. Red arrows point out knob-like protrusions, while cyan arrows highlight rougher protrusions on the cell surface. Additionally, yellow arrows indicate white dots that are predominantly distributed throughout the cell structure. Scale bar: 10 μm.

Compared to the morphology of undifferentiated classical monocytes (CD14^+^ CD16^low^) on 3D-printed PCL scaffolds (HT92, LT92) as shown in Figure 9, the differentiation toward Mo-DC was successful (Figure 10). When the morphology of Mo-DC was observed on scaffolds and 2D TCP, SEM images revealed that cells were attached on both (HT92 and LT92) scaffolds and 2D TCP, and cells portrayed a rounded morphology with dendritic protrusions. The protruding dendrites showed slightly variable characteristics between the different groups: in 2D TCP, Mo-DC showed typical dendrites with needle-like structure and ruffles in cell body (Figure 10, white arrow). Mo-DC on HT92 portrayed thicker and more knob-like protrusions (Figure 10, red arrow), while Mo-DC on LT92 depicted more elongated cell morphology with rougher protrusions on the cell surface (Figure 10, cyan arrow). Within the protrusions on HT92, white dots can be observed to occupy most of the cell structure (Figure 10, yellow arrow).

**Figure 11:**
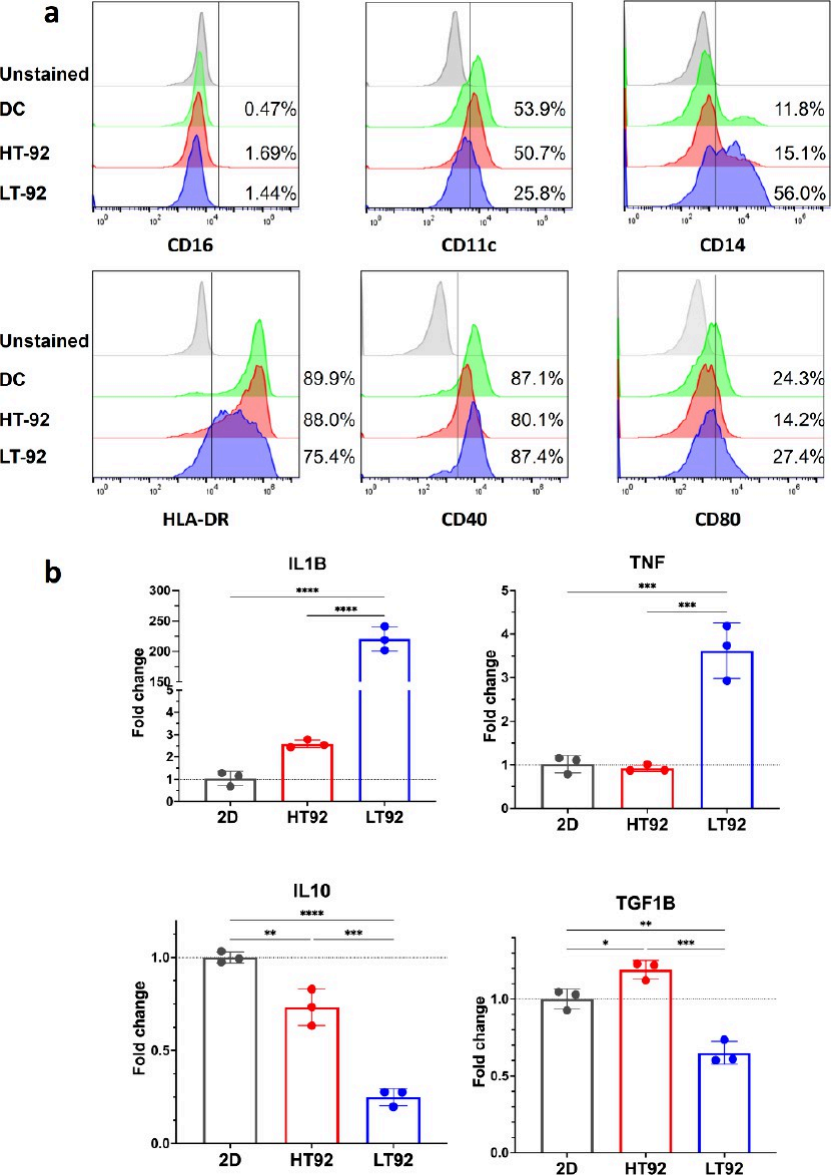
(a) *In vitro* differentiation of classical monocytes (CD14^+^ CD16^low^) into Mo-DC on tissue culture plates (TCP), HT92 and LT92 PCL porous scaffolds characterized by CD14, CD16, CD11, HLA-DR, CD80, and CD40 markers. (b) Gene expression of pro-inflammatory genes of IL1B and TNF-α and anti-inflammatory genes IL10 and TGF1B for Mo-DC cultured on HT92 and LT92 compared to TCP control. *N* = 3, *p < 0.05, **p < 0.01, ***p < 0.001, ****p < 0.0001.

Differentiation from classical monocytes (CD14^+^ CD16^low^) into Mo-DC was marked by downregulating CD14 expression (>80%, supplementary Figure S8) as shown by flow cytometry (Figure 11a). Slight differences in expression can be seen in DC cultured on LT92 compared with HT92 and 2D TCP. Mo-DC differentiated on LT92 had a higher expression of monocyte markers CD14 (56%) compared to HT92 and 2D TCP (15% and 11%, respectively). Expression of CD11 and HLA-DR was slightly downregulated in LT92 (25% and 75%, respectively) compared to HT92 scaffolds (50% and 88%, respectively). Activation markers CD80 and CD40 were highly expressed in LT92 (27% and 87%, respectively) compared to HT92 (14% and 80%, respectively), while being comparable to 2D TCP (24% and 87%, respectively).

Gene expression of pro-inflammatory genes IL1B (*p* < 0.0001) and TNF-α (*p* < 0.001) were significantly upregulated while expression of anti-inflammatory genes IL10 (*p* < 0.001 and *p* < 0.0001, respectively) and TGF1B (*p* < 0.001 and *p* < 0.01, respectively) were significantly downregulated in Mo-DC cultured on LT92 compared to HT92 and 2D TCP control (Figure 11b). Mo-DC cultured on HT92 had a comparable expression of pro-inflammatory genes IL1B and TNF-α to cells cultured on 2D TCP control with no significant differences. For anti-inflammatory markers, IL10 and TGF1B, significant differences were observed between Mo-DC on HT92 compared to 2D TCP, while HT92 showed lower IL10 (*p* < 0.01) and higher TGF1B (*p* < 0.05) than 2D TCP.

## Discussion

### Effect of NaCl- and NIPS-Based 3D Printing on Scaffold Microporosity

The objective of this study was to fabricate 3D printed porous PCL scaffolds tailored for bone tissue engineering with varying porosity and stiffness and to evaluate their impact on the proliferation and osteogenic differentiation of hBMSC, along with the activation of dendritic cells. Even though extrusion-based 3D printing of melted PCL can create scaffolds with large scale porosity (millimeters) by controlling the distance between the printed filaments, these filaments are usually solid and rigid.^19,21^ To address these challenges, a nonsolvent-induced phase separation (NIPS) printing technique was implemented. Deposition of the PCL-acetone viscous solution into ethanol yielded filaments characterized by high flexibility and porosity. As shown from the micro-CT analysis, NIPS resulted in interconnected microporosity smaller than 20 μm. The exchange of the nonsolvent and solvent between the ethanol bath and PCL filaments leads to an increase in the nonsolvent content in the filaments, inducing phase separation within the printed filaments. This resulted in the formation of a polymer-rich phase, which constitutes the PCL matrix, and a polymer-poor phase, which forms the micropores.^22−24^ Similar to this work, Kim et al., combined NIPS and 3D printing to produce PCL-based porous scaffolds.^25^ They used tetrahydrofuran as a solvent to 3D print PCL scaffolds with an overall porosity of 78.4 ± 1.2% and average micropore size of 2.8 ± 1.2 μm. Unlike in their work, acetone was used, which is a more sustainable and less hazardous polymer solvent.^26^ Apart from rinsing and sterilization, the produced scaffolds necessitated no additional postprinting treatment.

To further enhance the microscale internal porosity of both LT and HT printed filaments, a salt-leaching process was integrated with 3D printing by incorporating NaCl particles (40–90 μm) in the PCL-acetone mixture. In salt-leached scaffolds, it is widely noted that initial salt concentration directly influences overall porosity.^11^ In agreement with that, our micro-CT and SEM results confirmed that the salt concentration directly influenced the porosity. Our study demonstrated that higher concentrations of NaCl yielded higher porosity levels and faster salt removal after washing. The measured porosity of the HT23 group was lower than the theoretical value, suggesting the retention of some salt crystals within the filaments, likely due to lower initial salt concentrations. Qualitative observations by Jakus and colleagues indicated that increasing the salt content in PLGA led to accelerated salt leaching and increased interconnectivity. Consistent with these findings, our results demonstrate that, regardless of the printing temperature, a higher NaCl content in the PCL matrix not only accelerated salt leaching but also facilitated extensive salt removal from the PCL scaffolds. The removal of NaCl relies mainly on the water diffusion into the polymer matrix to dissolve and dissociate the salt particles.^11^ The PCL matrix acts as a barrier to water diffusion. The lower the salt concentration is, the more polymer around salt particles, as shown in our micro-CT images. Thus, increasing the salt concentration or inducing NIPS-based interconnected microporosity significantly enhanced salt removal due to the enhanced diffusion. Consequently, this led to higher open porosity and improved interconnectivity, closely aligning with the theoretically predicted porosity levels. Altogether, our modified printing approach represents an easy way to create highly porous structures without the use of hazardous solvents that require extensive washing procedures.

### Effect of NaCl- and NIPS-Based Microporosity on Mechanical Properties and Degradation

Naturally, bone has varying degrees of porosity, which directly influence their mechanical properties. Compact bone, characterized by its dense and rigid nature, exhibits high tensile stress tolerance (approximately 150 MPa) but limited strain capacity (around 3%) prior to fracture. Conversely, spongy bone, with its highly porous nature, can withstand lower stress levels (about 50 MPa) while allowing for significantly higher strain (approximately 50%) before failure.^27,28^ To mimic these mechanical properties, the porosity of scaffolds through modification of the printing process was adjusted. 3D printing of PCL at 120 °C without porosity (HT0 group) yielded rigid scaffolds with an elastic modulus (E) of 403 ± 19 MPa. Increasing salt concentration and subsequently the porosity resulted in a significant decrease in modulus of elasticity as well as the tensile stress of the HT92 group. Above its melting point (60–70 °C), PCL molecular segment motion is initiated, allowing for unrestricted movement of the polymeric chains. Upon rapid cooling postprinting, the polymer transitions to a semicrystalline state, featuring crystalline zones interspersed with rubbery amorphous entangled chains.^29^ This resulted in a more densely packed microstructure, leading to an increased filament stiffness.

On the other hand, by using NIPS-based printing, remarkably fabricated flexible scaffolds that can be easily folded and reversibly deformed under axial compressive loads. Moreover, this technique facilitated rapid 3D printing of PCL into substantial self-standing and anatomically precise structures, such as a sheep bone mandible measuring 3 cm in height. However, employing a combination of salt leaching- and NIPS-based printing resulted in decreasing the elasticity and lowering the strain value from 435 to 220% due to the decrease in the NIPS-based microporosity. Around RT, the amorphous phase of PCL is in its rubbery state.^29^ In NIPS-based printing, solvent molecules such as acetone diffuse between the polymer chains, and disrupt their intermolecular forces, enabling free movement of the chains within the solvent and facilitating shear-induced alignment with the printing direction as shown in this study (Figure S5).^30,31^ Unlike the rapid cooling in HT-based printing, solidification in ethanol occurs gradually, resulting in a more aligned fiber and microporous structure, thereby enhancing their ductility. It is worth noting that, in contrast to our simple method for generating highly flexible scaffolds, Jakus et al. employed a more complex approach involving a trisolvent mixture containing dichloromethane (as an evaporant), 2-butoxyethanol (as a surfactant), and dibutyl phthalate (as a plasticizer) to 3D print hyperplastic bone-like PCL-hydroxyapatite (HA) composites.^32^ Compared to our flexible NIPS-based scaffolds, these scaffolds demonstrated a lower tensile strain of 61.2 ± 6.4% and elastic modulus of 10.3 ± 1.3 MPa.^32^ Unlike our pure 3D printed PCL tensile samples, they utilized unprinted PCL-HA cast samples, which may account for the differences.

It is important to note that the high molecular weight PCL (Mn: 80 kDa) was not used for the HT printing due to technical challenges. Its increased viscosity made printing very difficult. Printing with this high molecular weight PCL required a slow speed (0.5 mm/s) and high printing pressures (8 bar) at temperatures above 140 °C, which led to rapid material degradation. Additionally, incorporating various concentrations of NaCl particles into the polymer significantly increased the viscosity, complicating the printing process compared to that of the neat polymer. When mixed with NaCl, the composite became nearly nonflowable and could not be extruded through 0.4 and 0.6 mm nozzles, resulting in clogging issues. Similarly, using low molecular weight PCL (Mn: 45 kDa) was unsuitable for low-temperature NIPS-based printing as it became brittle and crumbled easily during handling. Therefore, we opted for high molecular weight PCL for NIPS-based printing.

The degradation process of aliphatic polyesters like PCL involves the hydrolysis of labile ester bonds, with the rate of degradation primarily dependent on the accessibility of water to these bonds.^33^ The accelerated degradation test conducted in our study revealed the significant impact of porosity on the degradation of PCL scaffolds. The porosity induced by salt leaching in the HT scaffolds had a significant impact on the degradation. However, these large pores did not change the degradation profile of the LT scaffolds. This is highly likely due to the dominance of the small microporosity (more than 60% total porosity of the printed filament) in the LT scaffolds. In agreement with our findings, Zhang et al. observed that PCL scaffolds with higher porosities exhibited more pronounced weight loss.^34^ They suggested that the influence of porosity on degradation behavior was governed by both water permeability and hydrolysis effects: specifically, when water permeability prevailed, acid byproducts could efficiently diffuse outward, preventing acid accumulation. Consequently, higher porosity provided a larger surface area for hydrolysis, resulting in accelerated scaffold degradation rate.^34^

### Effect of NaCl- and NIPS-Based Microporosity and Stiffness on hBMSC

It is well-known that the porosity of scaffolds plays a significant role in bone formation *in vitro* and *in vivo*.^4,35^ Kuboki et al. demonstrated the importance of porosity in bone regeneration by utilizing a rat ectopic model by comparing solid and porous hydroxyapatite particles for BMP-2 delivery. They found that while no new bone formed on the solid particles, the porous scaffolds facilitated direct formation of mineralized tissue.^36^ The current study hypothesized that the large micropores due to the salt leaching (avg. 40 μm) method can offer enhanced support for hBMSC adhesion, whereas small micropores (avg. 10 μm) due to NIPS may stimulate osteogenic differentiation due to their large surface area and interconnectivity.^37−39^ In agreement with this hypothesis, on day 1, the large microporosity enhanced hBMSC attachment and spreading on HT92 and LT92 when compared to their counterparts, as shown in the live–dead images. However, the microporosity induced by NIPS or salt leaching, when compared to the nonporous HT0 group, was not the decisive factor for increasing cell proliferation. Particularly on days 1 and 7, the introduction of microporosity by salt leaching resulted in decreased cell number compared to HT0. A possible explanation is that the introduction of microporosity decreased the stiffness and slowed cell proliferation. It was reported that stiff substrates stimulate cells to form cytoskeleton stress fibers, thus leading to enhanced spreading, and proliferation.^40−42^ Moreover, this study hypothesized that macropores produced by the printing process (due to the distance between the filaments) may support cell proliferation, since large open spaces supply enough oxygen and nutrients.^4,43^ Our findings indicated that on day 14, there was no notable variance in cell proliferation and metabolic activity, affirming the capacity of the larger pores to sustain the nutrition and oxygenation essential for cellular growth. Taken together, the results obtained from the live–dead imaging, metabolic activity, and dsDNA quantifications confirmed not only the cytocompatibility of all scaffolds but also significant cell growth from 1 to 14 days. Moreover, these results indicate the complete removal of NaCl from the scaffolds as it was reported that the incomplete removal of CuSO_4_ from PLGA scaffolds resulted in cell death and lack of cell adhesion.^11^

Cells continuously sense their microenvironment and adapt over time to regulate various functions, including proliferation, migration, and differentiation.^44^ Additionally, it is widely accepted that both substrate porosity and stiffness can influence the osteogenic differentiation of MSC. These effects of substrate stiffness can persist even after cells are transferred to a different substrate.^4,45^ The ability of the printed scaffolds to support hBMSC differentiation was confirmed through RUNX2 expression, Collagen I production, ALP activity, and mineralization. Our findings revealed the complex interplay between porosity and stiffness in influencing the osteogenic differentiation of hBMSC. Osteogenic differentiation, marked by the production of RUNX2 and Collagen type-1, along with increased ALP activity and Alizarin Red staining, has been shown to be enhanced on stiffer substrates.^46,47^ Although the stiffer HT groups seemed to enhance the production of RUNX2 and Collagen I compared to the LT groups, the data from ALP activity and Alizarin Red staining do not fully confirm the role of stiffness in promoting osteogenic differentiation. This is because the addition of large microporosity in the HT92 scaffolds reduced stiffness but significantly increased ALP levels at days 1 and 7 compared to HT0. In contrast, the large microporosity induced by salt leaching decreased the ALP activity in LT92. Interestingly, NIPS-based microporosity in the highly flexible LT0 group demonstrated the highest ALP levels over the culture time, suggesting that the influence of the high level of small microporosity (total filament porosity >60% and pore size < 20 μm) may outweigh the impact of scaffold stiffness. However, examination of the mineralized matrix using Alizarin red staining indicated that LT0 exhibited the lowest level of mineralization. The salt leached large microporosity (40 μm) increased the levels of the mineralization in LT92 and HT92. A possible speculation is that small microporosity (10 μm) may be necessary for initial stages of osteogenic differentiation, like increased ALP activity, while larger microporosity (40 μm) may influence later osteogenic processes such as matrix mineralization. Nevertheless, in the literature, it is difficult to identify the optimal porosity (both total porosity and pore size) and stiffness values for hBMSC proliferation and osteogenic differentiation due to variations in scaffold materials, shapes, surface alterations, fabrication techniques, and culture conditions in the published reports.^4,48^

### Effect of NaCl- and NIPS-Based Microporosity and Elasticity on Mo-DC

Implantation of scaffolds in the bone defect results in immune responses that in turn, influence the immune-mediated bone tissue engineering. The evidence is piling up that dendritic cells play a vital role in bone regeneration by contributing to the intricate interplay between the immune system and regenerative processes.^49^ Scaffold porosity and stiffness were reported to have great impacts on the phenotype and function of innate immune cells especially macrophage polarization.^2^ However, there is little information about the effect of physicochemical properties of 3D printed PCL scaffolds on the fate of the innate immune cells, DC. Chen et al. investigated the role of 20, 40, and 90 μm pores of PDMS substrates on the *in vitro* maturation of DC and reported an enhanced maturation of the cells as pore size decreased.^10^ In the current study, cells were cultured on a tissue culture plate (TCP) as a standard control. Cell culture plastics are very stiff with Young’s modulus more than 1 GPa.^50^ The only significant difference between HT92 and TCP was a significant decrease in IL10 mRNA and a significant increase in TGF1B mRNA expression, which could indicate an altered anti-inflammatory response of Mo-DC cultured on HT92. HT92 scaffolds with overall lower total microporosity (due to only salt leaching) do not seem to have large effects on Mo-DC differentiation and activation, while LT92 scaffolds with smaller NIPS-based micropores and lower stiffness showed retained expression of monocyte markers which might indicate less differentiation into Mo-DC. On the other hand, Mo-DC cultured on LT92 scaffold showed significant changes in gene expression of pro-inflammatory (IL1B and TNF) and anti-inflammatory (IL10 and TGF1B) cytokines compared to both TCP and HT92 scaffold. This suggests a pro-inflammatory response of Mo-DC cultured on LT92. These differences might be due to reduced stiffness and the increased roughness of the LT92 scaffold as shown in SEM images. Chakraborty et al. compared mouse bone marrow derived DC grown *in vitro* on soft substrates (E = 2 kPa) and stiff substrates (E = 50 kPa) and reported markedly enhanced production of inflammatory cytokines characterized by upregulation of IL1β and TNF-α in DC grown *in vitro* on stiff substrates.^51^ In addition to the use of human cells in our study, the modulus of elasticity (E) values for HT92 and LT92 PCL scaffolds were 143 ± 2 and 28 ± 4 MPa respectively, with several orders of magnitude higher than their PDMS substrates, thus providing a possible explanation for the differences between the two studies.

In summary, it was shown that the approach of combining different fabrication methods can produce highly porous and cell-friendly scaffolds at clinically significant scales and shapes using entirely safe, established thermoplastic polymers. The osteogenic performance and immunomodulatory potential of such scaffolds can make them considerably more efficient and cost-effective approach for bone tissue engineering compared to the conventional bone grafting techniques.^2,32^

The field of tissue engineering and regenerative medicine is increasingly moving toward personalized approaches, taking into account not only patient variations but also the specific requirements of different tissues. For example, natural bone has heterogeneous stiffness, with compact bone being stiffer and less flexible than cancellous bone. In cases involving infants with growing bones, where more flexible and degradable scaffolds are required, or in bone defects located in cancellous regions with low mechanical loads, LT-based scaffolds may be appropriate. Conversely, for defects in load-bearing areas such as long bones, a combination of LT and HT scaffolds could be used to mimic the dense, rigid outer compact bone and the flexible, spongy inner bone. However, since the scaffolds in this study are made of PCL, a material known to induce foreign body reactions, both types, especially LT - require further modification and investigation to improve their performance for more immune-mediated bone regeneration.

## Conclusions

PCL scaffolds were successfully fabricated at high and low temperatures with different porosities, elasticity, and degradation using a combination of extrusion-based printing and salt leaching techniques with and without nonsolvent induced phase separation (NIPS). The interplay between porosity and stiffness was employed to evaluate the influence of these two parameters on hBMSC and DC. The interactions between hBMSC and scaffolds suggested that the significant effect of these two parameters on cell proliferation and osteogenic differentiation was more profound at early time points. Within the porosity and stiffness range of the current study, scaffolds printed at low temperature with high microporosity and low stiffness produced more mineralization but triggered a pro-inflammatory response of DC. Collectively, these results suggest that our modified 3D printing approach holds promises in producing cell-instructive polymeric scaffolds with tailored porous architecture, flexibility, and degradation rate for bone tissue engineering applications.

## Supporting Information Available

Additional experimental details and methods, including calculations for microporosity, scaffold shrinkage and swelling, along with images illustrating porosity directionality, cell viability, and flow cytometry analysis (PDF)
