# Supplementary material for: Impact of Porosity and Stiffness of 3D Printed Polycaprolactone Scaffolds on Osteogenic Differentiation of Human Mesenchymal Stromal Cells and Activation of Dendritic Cells
Source: ACS Biomater Sci Eng. 2024 Nov 1;10(12):7539–54. doi: 10.1021/acsbiomaterials.4c01108 (PMC11632652; doi:10.1021/acsbiomaterials.4c01108)
Supplement: Supplementary file 1 — ab4c01108_si_001.pdf [file ab4c01108_si_001.pdf]

Supplementary Data

Impact of Porosity and Stiffness of 3D Printed Polycaprolactone Scaffolds on Osteogenic Differentiation of Human Mesenchymal Stromal Cells and Activation of Dendritic Cell

Mehmet Serhat Aydin[1], Nora Marek[1], Theo Luciani[1], Samih Mohamed-Ahmed[1], Bodil Lund[2,3], Cecilie Gjerde[1], Kamal Mustafa[1], Salwa Suliman[1*], Ahmad Rashad[1*]

[1]Center of Translational Oral Research (TOR), Department of Clinical Dentistry, University of Bergen, Bergen 5009, Norway.
[2]Department of Dental Medicine, Karolinska Institute, Stockholm 17177, Sweden.
[3]Medical Unit of Plastic Surgery and Oral and Maxillofacial Surgery, Karolinska University Hospital, Stockholm 17177, Sweden.
[4]Bioengineering Graduate Program, Aerospace and Mechanical Engineering, University of Notre Dame, Notre Dame, Indiana 46556, United States.

[†]These authors contributed equally to this work.

[*]Authors to whom any correspondence should be addressed.

Email: Kamal.Mustafa@uib.no, Salwa.Suliman@uib.no and ahmad.elsebahy@uib.no

Experimental Porosity Evaluation

The microporosity, introduced by nonsolvent induced phase separation (NIPS) and salt leaching methods, was calculated from the weights and volumes of scaffolds. The average microporosity within the filaments of three scaffolds was determined by calculating the amount of water absorbed, derived from the difference between the wet and dry weights of the scaffold. Details and formulas are given below.

$$Volume\ of\ scaffold\ (all\ filaments)\ V_{\sigma}^{s} = Volume\ of\ PCL\ (V_{PCL}^{s}) + Volume\ of\ liquid\ absorbed\ (V_{l}^{s})$$

$$V_{\sigma}^{s} = V_{PCL}^{s} + V_{l}^{s}$$

$$Microporosity\ (\emptyset_{\mu}) = 1 - \frac{Volume\ of\ PCL}{Volume\ of\ scaffold} = 1 - \frac{V_{PCL}^{s}}{V_{\sigma}^{s}}\ or\ \frac{Volume\ of\ liquid}{Volume\ of\ scaffold} = \frac{V_{l}^{s}}{V_{\sigma}^{s}}$$

$$\emptyset_{\mu}(LT) = \frac{Volume\ of\ liquid}{Volume\ of\ PCL + Volume\ of\ liquid}$$

$$\emptyset_\mu(in\,filament,LT) = \frac{w_l^s}{w_{PCL}^s + w_l^s} \,º\, \frac{V_l^s}{V_{PCL}^s + V_l^s} \quad (1) \rightarrow E1$$

Here $V_l^s and\, V_{PCL}^s$ seem to be unknown but they can be calculated through weight and density relation.

$$W_{wet}^s - W_{dry}^s = w_l^s = V_l^s \, x \, \rho_l$$

$$V_l^s = \frac{w_l^s}{\rho_l} \ldots(2)$$

$$V_{PCL}^s = \frac{w_{PCL}^s}{\rho_{PCL}} \ldots(3)$$

Substituting equation (2) and (3) into (1) yields equation (4) and rearranging the equation (4) becomes equation (5) below.

$$\emptyset_\mu = \frac{\frac{w_l^s}{\rho_l}}{\frac{w_l^s}{\rho_l} + \frac{w_{PCL}^s}{\rho_{PCL}}} = \frac{1}{1 + \frac{w_{PCL}^s}{w_l^s} x \frac{\rho_l}{\rho_{PCL}}} \ldots(5)$$

This formula (5) indicates that microporosity depends on both the ratio of the dry weight of the scaffold to the weight of liquid absorbed by the scaffold, as well as their density ratio. The formula can be expressed differently, leading back to equation (1), which is identical.

$$\emptyset_\mu = \frac{1}{1 + \frac{\frac{w_{PCL}^s}{\rho_{PCL}}}{\frac{w_l^s}{\rho_l}}} = \frac{1}{1 + \frac{V_{PCL}}{V_l}} \ldots(6)$$

Calculations of Dual Microporosity in NIPS-Based 3D Printed Scaffolds

Combining NIBS and slat leaching resulted in creating dual microporosity with two distinct pore formation mechanisms. NIPS creates micropores with small volume (V_{NIPS}^s), while salt leaching by NaCl introduces micropores with larger volume (V_{NaCl}^s). Since NaCl is not soluble in ethanol, NaCl-based porosity is not induced in ethanol and the NaCl particles occupy the

volumes that eventually will form the (V^s_{NaCl}). Therefore, the weight of wet scaffolds in ethanol is sum of weight of PCL itself, weight of ethanol filling NIPS pore sites (V^s_{NIPS}) and weight of NaCl (Equation 7a).

$$W^s_{EtOH} = w^s_{EtOH,NIPS} + w^s_{NaCl} + w^s_{PCL} \ldots(7a)$$

As soon as the scaffolds come into contact with water during the washing process, the NaCl particles dissolve, leaving pores behind. These pores are rapidly filled with water once the leaching process is complete. Hence, the weight of wet scaffolds in DI water comprises the combined weight of water at NIPS-pores sites, water at NaCl-sites, and the weight of PCL itself (Equation 8a) as shown in Supplementary Figure 1.

$$W^s_{H2O} = w^s_{H2O,\,NIPS} + w^s_{H2O,\,NaCl} + w^s_{PCL} \ldots(8a)$$

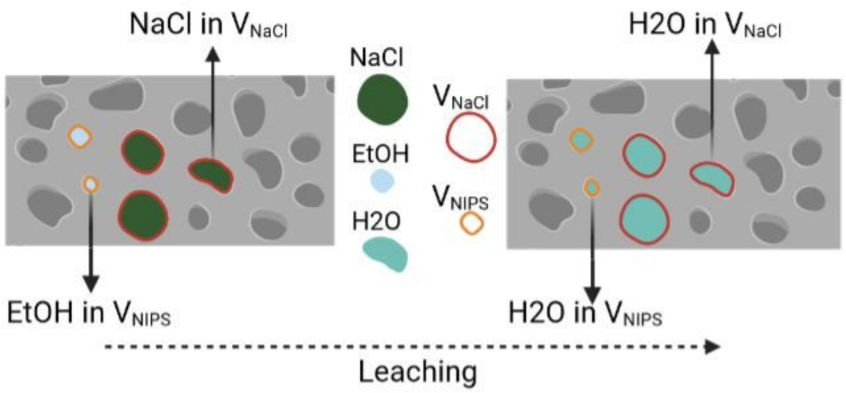

Figure S1. Schematic illustration of formation mechanism of NIPS- and NaCl-based microporosity.

After the scaffolds were thoroughly dried, the residual weight is solely attributed to PCL (Equation 9a).

$$W^s_{Dry} = w^s_{PCL} \ldots(9a)$$

The equations 7a, 8a, 9a can be rewritten based on $m = \rho \, x \, V$ relation as follows,

$$W^s_{EtOH} = \rho_{EtOH} \times V^s_{NIPS} + \rho_{NaCl} \times V^s_{NaCl} + \rho_{PCL} \times V^s_{PCL} \ldots(7b)$$

$$W^s_{H2O} = \rho_{H2O} \times V^s_{NIPS} + \rho_{H2O} \times V^s_{NaCl} + \rho_{PCL} \times V^s_{PCL} \ldots(8b)$$

$$W^s_{Dry} = \rho_{PCL} \times V^s_{PCL} \ldots(9b)$$

By subtracting the equation 9b from the 7b and from the 8b, respectively and simultaneously gives the 2 equations below.

$$W^s_{EtOH} - W^s_{Dry} = \rho_{EtOH} \times V^s_{NIPS} + \rho_{NaCl} \times V^s_{NaCl} \ldots(10)$$

$$W^s_{H2O} - W^s_{Dry} = \rho_{H2O} \times V^s_{NIPS} + \rho_{H2O} \times V^s_{NaCl} \ldots(11)$$

A system of linear equations having 2 equations, 2 unknowns (equation 10 and 11) was obtained. Roots of the system $V^s_{NIPS}\, and\, V^s_{NaCl}$ can be found by solving equations simultaneously with any computational tool (i.e, excel).

$$\begin{bmatrix} W^s_{EtOH} - W^s_{Dry} \\ W^s_{H2O} - W^s_{Dry} \end{bmatrix} = \begin{bmatrix} \rho_{EtOH} & \rho_{NaCl} \\ \rho_{H2O} & \rho_{H2O} \end{bmatrix} \times \begin{bmatrix} V^s_{NIPS} \\ V^s_{NaCl} \end{bmatrix} \ldots(12) \rightarrow E2$$

Two equations can be used to calculate to find individual induced porosity by either NaCl or NIPS respectively. In addition, porosity percentage of salt leaching and NIPS within scaffold volume can also be calculated as follows,

$$\emptyset^s_{NaCl} = \frac{V^s_{NaCl}}{V^s_{NaCl} + V^s_{NIPS} + V^s_{PCL}} \quad (13)$$

$$\emptyset^s_{NIPS} = \frac{V^s_{NIPS}}{V^s_{NaCl} + V^s_{NIPS} + V^s_{PCL}} \quad (14)$$

Calculations of NaCl-Based Porosity in HT 3D Printed Scaffolds

$$Volume\ of\ scaffold = Volume\ of\ PCL + Volume\ of\ NaCl\ leached$$

$$V^s_{\sigma} = V^s_{PCL} + V^s_{NaCl}$$

$$Microporosity = \frac{Volume\ of\ NaCl\ Leached}{Volume\ of\ scaffold}$$

$$\emptyset_{\mu}(in\ filament) = \frac{V_{NaCl}^{s}}{V_{PCL}^{s} + V_{NaCl}^{s}}(15) \rightarrow E3$$

Shrinkage, Swelling and Printability of LT NIBS-Based 3D Printed Scaffolds

Scaffolds (15 mm × 15 mm) with 4 layers and a 1.5 mm gap between strands were imaged using a stereomicroscope (Leica M205C Wetzlar, Germany), and analyzed with ImageJ software. The printability value was calculated from pore area and perimeter of 25 pores by employing the following equations:

$$Df_r = \frac{A_t - A_a}{A_t} x100\ (\%)\ (16)$$

$$P_r = \frac{L^2}{16A_p}(17)\ (2)$$

Where $A_t\ and\ A_a$ are the theoretical and actual area of the pore, respectively. L and A_p are the actual perimeter and area of a pore of a scaffold, respectively in the equation E2. For a perfect square pore, the printability (Pr) is 1.0. While there was no significant statistical difference between the scaffold groups, it was observed that the addition of NaCl slightly improved the printing quality as shown **Figure S2(a)**.

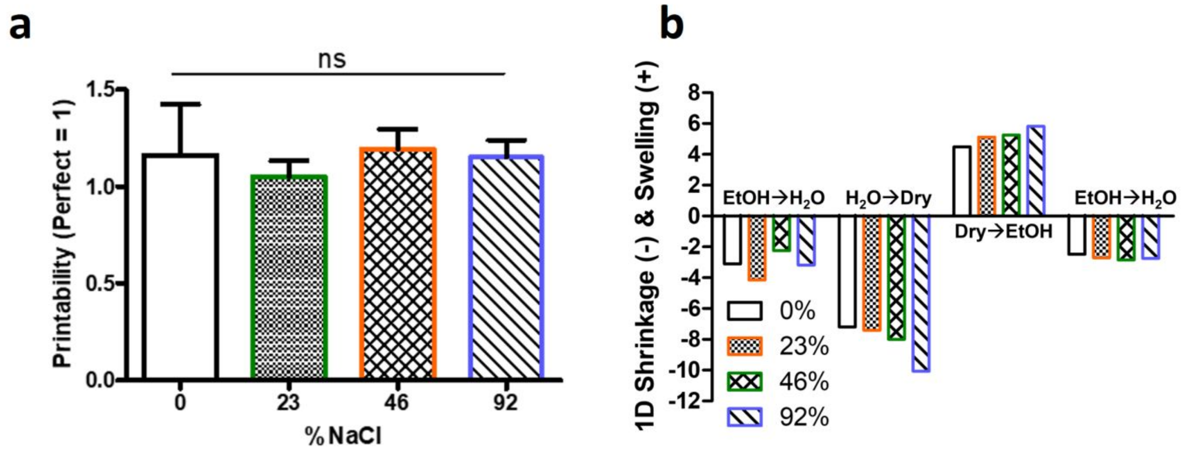

Figure S2. (a) Printability and **(b)** 1D shrinkage and swelling characteristics of NIPS-based printed scaffolds with various NaCl content (0, 23, 46, 92% w/w).

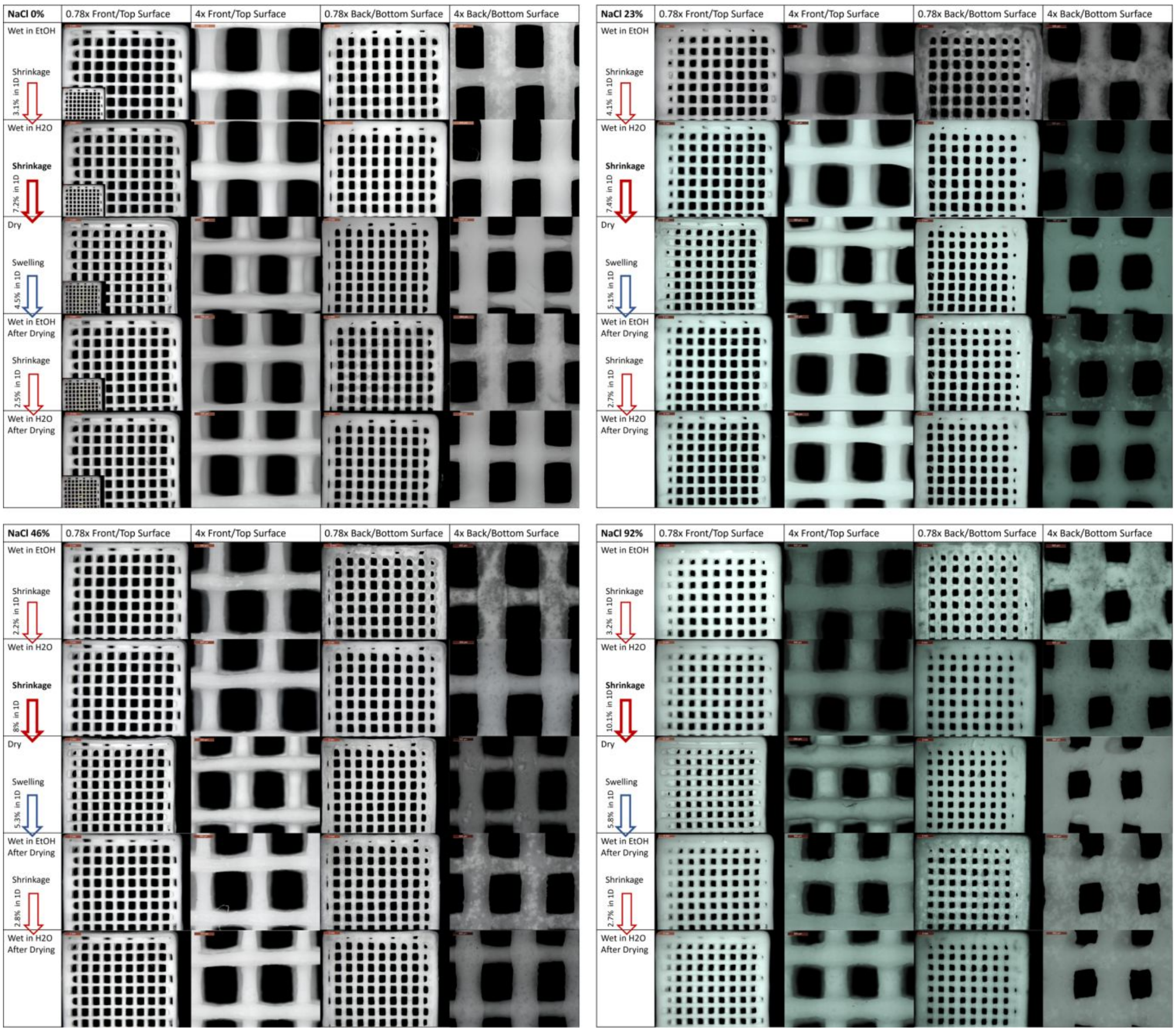

Figure S3. Shrinkage and swelling (%) of LT 3D printed scaffolds after soaking in different solutions. Scaffolds were printed in ethanol, moved to water, dried in air, and soaked in ethanol

and water again. Stereomicroscope images were taken for every condition and ImageJ analysis was performed to evaluate the dimensions stability of the scaffolds.

To evaluate the dimensions stability of the printed scaffolds after printing in ethanol, washing in water, drying in air, and wetting again for cell culture experiments, we analyzed the change in the length of scaffolds (1D change) as shown in **Figure S2(b).** The changes in length, representing 1D shrinkage and swelling indicated as red and blue arrows in **Figure S3**. All numerical values of the change in the length are presented in **Table S1.**

Table S1. LT scaffolds' parameters in terms of length and % shrinkage and swelling.

**Liquid**	**0% NaCl**	**23% NaCl**	**46% NaCl**	**92% NaCl**
	**Length printed scaffold**			
**In EtOH**	15.29±0.01	15.26±0.03	15.15±0.02	15.42±0.03
**In H_2O**	14.81±0.04	14.63±0.06	14.81±0.01	14.93±0.07
**Dry**	13.75±0.05	13.54±0.06	13.62±0.09	13.43±0.06
**In EtOH after**	14.37±0.07	14.24±0.13	14.34±0.06	14.21±0.07
**In H_2O after**	14.01±0.09	13.85±0.19	13.93±0.04	13.82±0.02
	**Shrinkage/swelling %**			
**EtOH to H_2O**	-3.1	-4.1	-2.2	-3.2
**H_2O to Dry**	-7.2	-7.4	-8.0	-10.1
**Dry to EtOH**	4.5	5.1	5.3	5.8
**H_2O to EtOH**	-2.5	-2.7	-2.8	-2.7

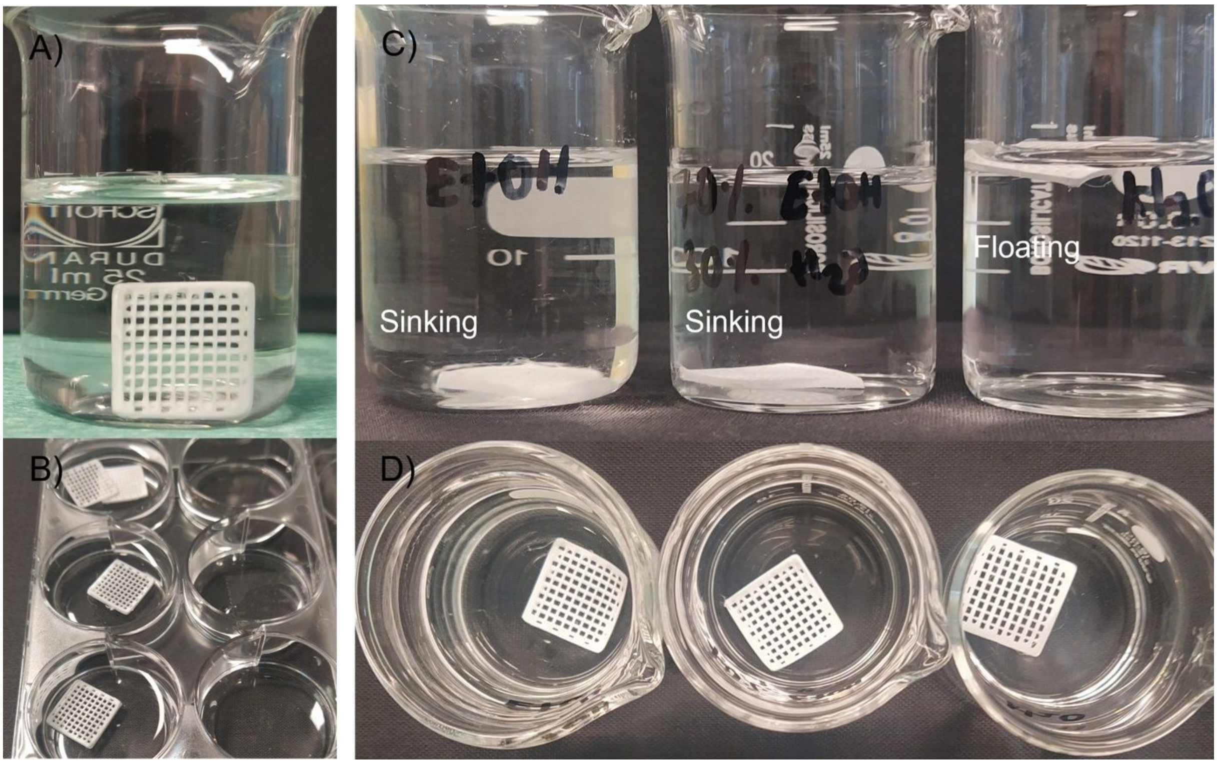

Figure S4. Photographs of 3D porous PCL LT scaffold in different solutions. Scaffold sinks before drying them out completely **(a)** side view and **(b)** top diagonal view; Scaffolds have different behavior depending on the solution after drying them completely. **(c)** Scaffolds at different liquids pure ethanol, 70-30% ethanol-water mixture, and pure water from left to right. The scaffolds sink in ethanol rich liquids whereas the third one floats in pure water. **(d)** Same beakers and scaffolds system from top view.

Effect of Printing on Directionality of the Microporosity

To evaluate the effect of printing direction on the orientation of the microporosity within the deposited filament, high resolution micro-CT analysis was conducted (**Figure S5**). At transverse cross section, pores and fibers were found to be random, whereas at coronal and sagittal cross sections pore orientation was along with printing direction in either 0° or 90° degrees respectively. This can be one of the reasons behind the high tensile strength, elongation, and flexibility of the NIPS-based scaffolds.

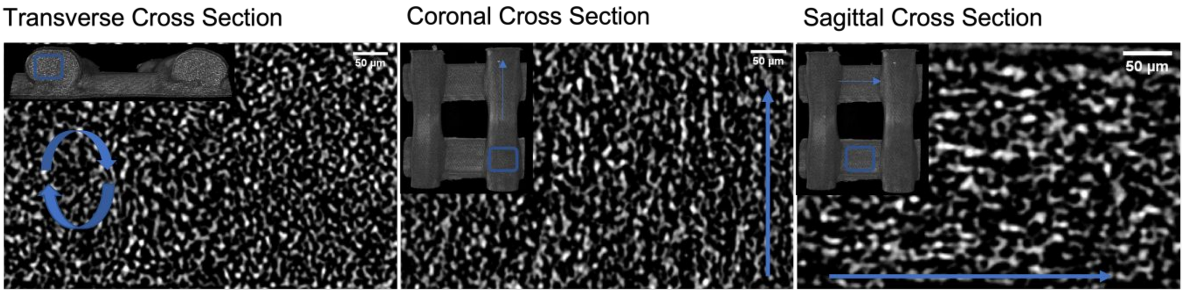

Figure S5. Micro-CT images at different sections showing the effect of printing direction on pore orientation in NIPS in LT printing.

Cell Viability of LT and HT scaffolds

All 3D printed scaffolds varying NaCl content (0%, 23%, 46%, 92% wt./wt.) displayed good biocompatibility, as confirmed by live-dead imaging and cell morphology images (Figure S6-S8) and showed no signs of cytotoxicity. NaCl percentage has no obvious impact on cell viability. However, cells seeded on porous scaffolds compared to non-porous HT0 scaffolds show early spreading and stretching and networking even at very early time point. Nevertheless, variations in cell morphology were observed among the groups at different time points.

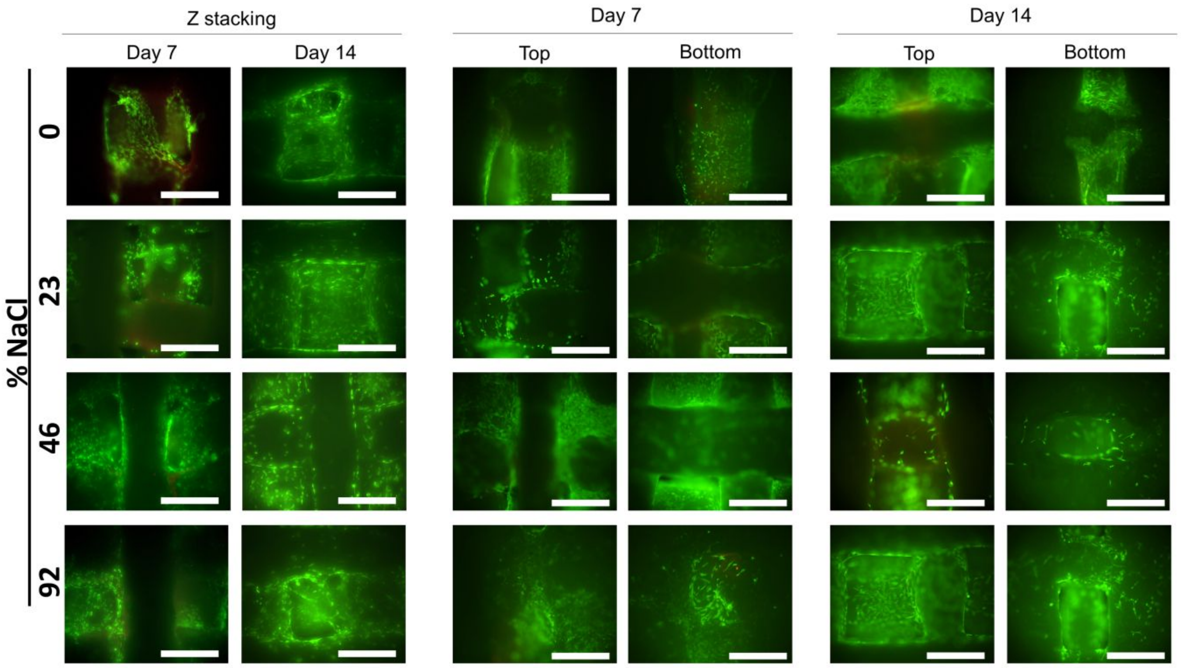

Figure S5. Fluorescence images of live/dead stain of LT 3D printed scaffolds. Regardless of the NaCl percentage, cells demonstrated excellent cell viability.

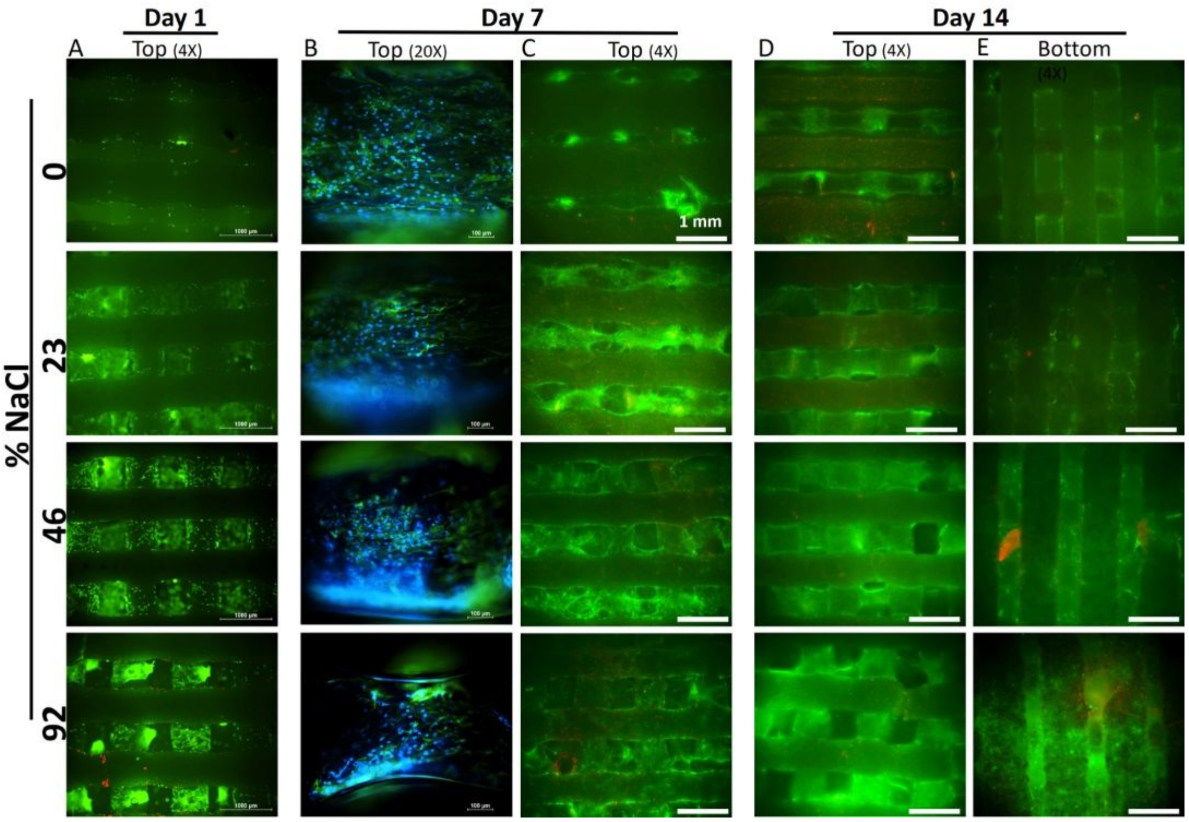

Figure S6. Fluorescence images of live/dead stain of HT 3D printed scaffolds. Regardless of the NaCl percentage, cells demonstrated excellent cell viability. DAPI (blue) and Phalloidin (green) staining demonstrated that, on day 7, cells seeded on porous scaffolds spread more compared to non-porous HT0 scaffolds.

Monocyte Isolation and Characterization

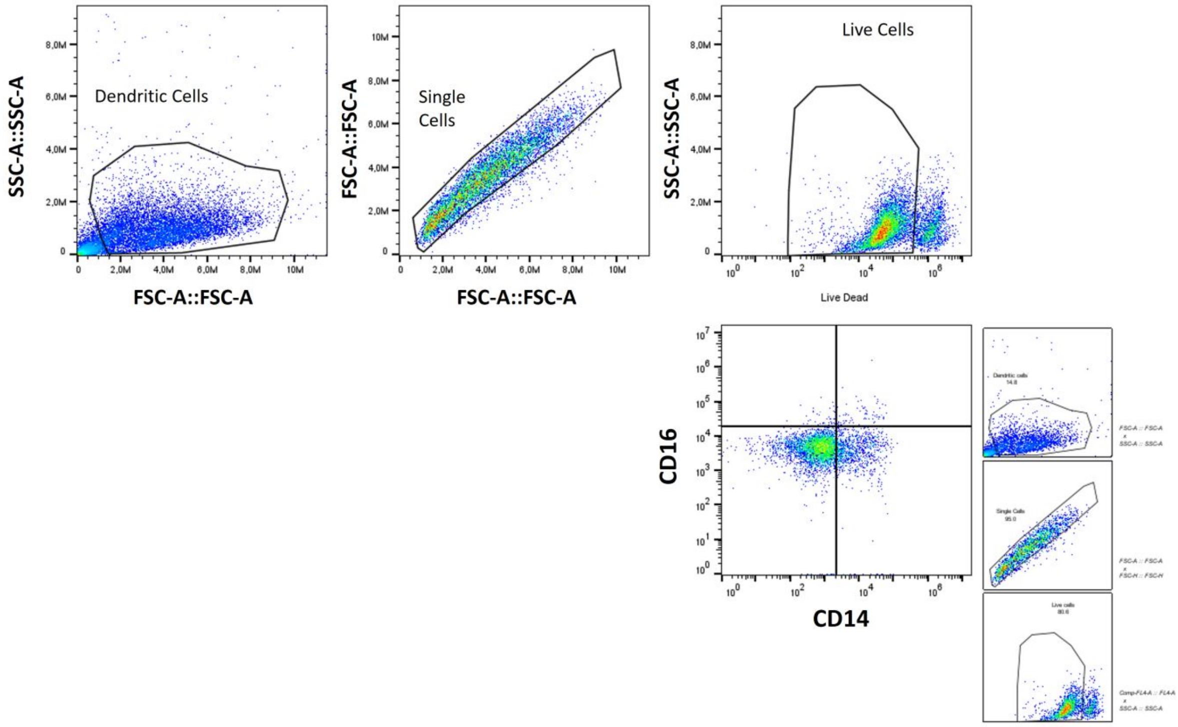

Figure S7. Gating strategy used in flow cytometry analysis. Shown are representative dot plots showing the gating strategy for monocyte-derived dendritic cell population, single cells, live/dead determination, and CD14 and CD16 subsets.

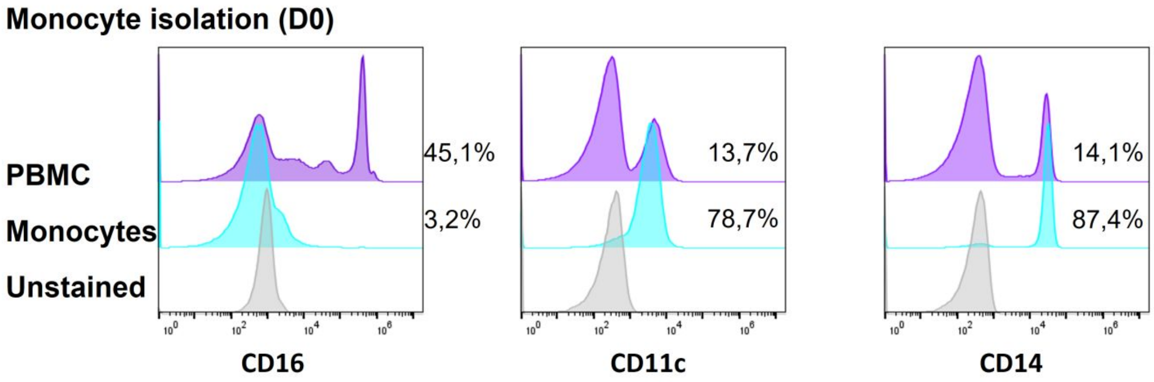

Figure S8. The purity of classical monocytes ($CD14^{+}CD16^{low}$) isolation. Flow cytometric analyses of purity of $CD14^{+}CD16^{low}$ monocytes after negative selection compared to PBMC (Peripheral Blood Mononuclear Cells). Cells expressing CD16 (left), CD11c (middle) and CD14 (right).
